# Transcriptional readthrough precedes alternative splicing programs triggered in CML cells by imatinib

**DOI:** 10.1126/sciadv.aea2475

**Published:** 2026-03-20

**Authors:** Paulina Podszywałow-Bartnicka, Morgan Shine, Jing Lin, Karla M. Neugebauer

**Affiliations:** Department of Molecular Biophysics and Biochemistry, Yale University School of Medicine, New Haven, CT, USA.

## Abstract

Cellular stresses regulate transcriptional readthrough, whereby RNA polymerase II elongates past a gene’s polyadenylation cleavage site without RNA cleavage. Readthrough has been reported in several cancer types. Here, we use long-read sequencing of nascent RNA to quantify transcriptional readthrough in chronic myeloid leukemia (CML) cells and characterize early responses to the targeted therapeutic, imatinib. We show that the amount, length, and gene specificity of readthrough increase within 1 hour, before gene expression and alternative splicing alterations emerge. Notably, imatinib-dependent messenger RNA (mRNA) isoform changes involved “readthrough chimeras,” in which exons from an upstream gene are alternatively spliced to exons in a downstream gene. Altered mRNA isoforms and chimera levels were detected in imatinib-resistant K562 cells as well as cells of patients with CML. Thus, imatinib can provoke a cascade of early changes to transcription and splicing fidelity that may lead to longer-term adjustments in gene expression, cancer cell differentiation, and the development of therapy resistance.

## Teaser

Precision RNA sequencing revealed the earliest response of leukemia cells to targeted therapy: transcriptional readthrough.

## INTRODUCTION

From prokaryotes to eukaryotes and from yeast to man, nascent RNA is the substrate of numerous RNA processing steps, including 5′-end capping, splicing, and 3′-end cleavage (*1*). Specific RNA sequences recruit the corresponding machineries—namely, the capping enzymes, the spliceosome, and the cleavage and polyadenylation complex (CPC)—that act quickly to modify the nascent transcript as it emerges from RNA polymerase II (Pol II). Human genes harbor an average of eight introns each, of which ~75% are cotranscriptionally removed by the spliceosome (*1*–*4*). The recent introduction of long-read sequencing of nascent RNA as a tool for analyzing cotranscriptional RNA processing has enabled researchers to associate multiple RNA processing steps with each other and with the position of Pol II. Emerging evidence points to tight coordination of intron removal at the level of individual transcripts (*5*–*8*). For example, unprocessed nascent transcripts—with all introns retained and a lack of cleavage at the polyadenylation site (PAS)—were observed in budding and fission yeasts, showing that RNA splicing and cleavage efficiencies are controlled at the individual transcript level. When 3′-end cleavage fails, Pol II proceeds past the PAS and continues downstream of the parent gene (*6*, *9*, *10*). A simple interpretation of this phenotype is that the activity of CPC is either delayed or blocked when a transcript has not been cotranscriptionally spliced. The consequences of transcriptional readthrough are the subject of intense investigation.

Numerous studies have identified transcriptional readthrough as a common, rapid response to cellular stress. Hypoxia, heat shock, starvation, and viral infection each lead to increases in the level of intergenic transcription, as measured by RNA sequencing (RNA-seq) experiments that identify “downstream of genes” (DoG) RNAs (*11*, *12*). Increased intergenic transcription can encompass many kilobases (kb) past the most distal gene boundary and is believed to be contiguous with the upstream “parent” gene rather than due to activation of downstream transcription start sites (TSSs) (*13*, *14*). When transcription continues into the next gene on the DNA strand, transcription and/or splicing of the “read-in gene” are often disrupted (*10*, *15*, *16*). Although DoG RNAs were initially considered to be stress induced, their presence has since been broadly documented in normal tissues, cancer cells, and aging cells and tissues (*17*–*19*). Transcriptional readthrough is a hallmark of clear cell renal carcinoma cells and prostate cancer cells, where a marked increase in transcription downstream of genes leads to the formation of chimeric RNA transcripts composed of exons from the parent gene spliced together with exons from the read-in gene (*20*, *21*). Chimeric transcripts can encode truncated proteins, freshly fused together functional globular domains, or, alternatively, proteins with intrinsically disordered extensions (*19*). Thus, the induced expression of chimeric transcripts and encoded proteins in response to stress may be physiologically relevant.

Transcriptional readthrough has been vastly understudied in the context of cancer. By contrast, pre-mRNA splicing regulation is often disrupted in cancer and even drives cancer initiation in some blood cancers, making splicing a target for clinical therapies (*22*). We are interested in the possibility that transcriptional readthrough and associated changes in RNA processing play roles in the development and treatment of chronic myeloid leukemia (CML), where alternative splicing has been identified in patients (*23*). Activation of cellular stress responses is detected in stem cells and accompanies carcinogenesis (*24*, *25*). These evolutionarily conserved pathways are detected in primary cells and hijacked by different cancer types, supporting survival and chemotherapy resistance. In CML, the unfolded protein response and phosphorylation of eIF2α accompany cancer progression and reduce the effectiveness of the targeted therapeutic drug imatinib (*26*, *27*). The activity of Bcr-Abl1 onco-kinase affects protein translation and induces stress granules in CML cells (*28*, *29*). Similar cellular phenotypes were detected in acute myeloid leukemia (AML), linking condensation of stress granules to changes in splicing regulation (*30*). Two chemotherapeutic agents used to treat AML, among other cancers, JTE-607 and camptothecin, cause transcriptional readthrough by directly inhibiting the enzymatic activity of the CPC and topoisomerase 1, respectively (*31*–*34*). These chemotherapeutic compounds directly and indirectly affect transcription and pre-mRNA splicing (*35*–*37*), making it unclear whether transcriptional readthrough is purely related to the biochemical activities of these drugs or, alternatively, whether it is related to the stress induced in cancer cells by therapeutic treatment.

Here, we have specifically addressed the possibility that transcriptional readthrough could be induced by therapeutic targeting of a cytoplasmic pro-oncogenic protein, Bcr-Abl1. We used imatinib treatment of CML cells as a well-studied model and assayed the cellular response to exposure times (1 and 18 hours), within one cell cycle period. Because imatinib directly inhibits the activity of BCR-Abl1 onco-kinase and not the transcription or RNA processing machinery, our experiment enables us to determine the transcriptional effects of cancer cells stressed by targeted cancer therapy. To achieve this, we have also implemented long-read sequencing of nascent RNA together with short-read sequencing datasets to quantify and rigorously characterize the occurrence of transcriptional readthrough at the gene and single-transcript level, enabling us to detect associated events like alternative splicing and changes in the formation of chimeric transcripts. The early response of CML cells to imatinib is not well characterized. Our approach detects some of the long-term alterations characteristic of month-long treatment that arise already within these short treatment periods. Together, our findings show that transcriptional readthrough is the first detectable effect of imatinib on gene expression, which precedes and likely facilitates later gene expression changes.

## RESULTS

### The targeted chemotherapeutic imatinib induces transcriptional readthrough in K562 cells

**Fig. 1.**
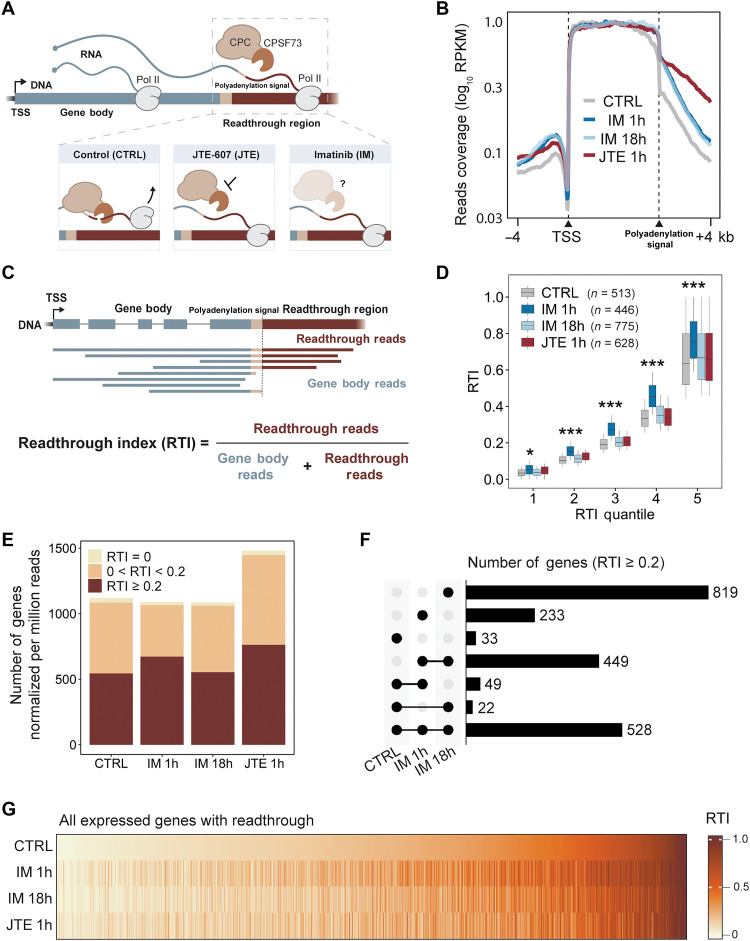
Imatinib treatment induces transcriptional readthrough detected by long-read sequencing. (**A**) Schematic showing transcriptional readthrough, which occurs upon impaired 3′-end cleavage. JTE-607 is a chemical inhibitor of CPSF73, and imatinib is a targeted therapeutic that inhibits the Bcr-Abl1 onco-kinase. (**B**) Metagene analysis of RNA-seq profiles for control (CTRL) and treated (IM 1h, IM 18h, and JTE 1h) cells. The *y* axis represents the read coverage combined from three biological replicates and analyzed in 40-nt bins. RPKM, reads per kilobase per million mapped reads. (**C**) Schematic representation of transcriptional RTI calculation. (**D**) Distribution of RTI values per gene for each condition presented for quantiles. One-way analysis of variance (ANOVA) with Dunnett’s test was used to compare impact of IM 1h to other conditions (***P < 0.00001, *P = 0.002); *n*, number of genes in a quantile. (**E**) Fraction of genes with more or less than 20% of long reads displaying readthrough (RTI = 0 in beige; RTI < 0.2 in yellow; RTI ≥ 0.2 in maroon). In (D) and (E), genes were required to have ≥10 reads per replicate to be included in these analyses. (**F**) Upset plot comparing genes with RTI ≥ 0.2 in CTRL, IM 1h, and IM 18h cells. (**G**) Heatmap of RTI values for each expressed gene [*n* = 1817 genes from (E)], ordered by RTI value in CTRL.

To study the effects of imatinib on RNA processing, we chose the K562 cell line, which originates from a patient with CML in blast crisis (*38*), as a model system. Transcriptional readthrough is typically studied by short-read sequencing of nuclear RNA (*34*). In this study, we implemented long-read sequencing of nascent RNA to detect and quantify the number and length of reads that extend past the PAS (Fig. 1A). Short-time (1 hour) imatinib treatment increased coverage within the intergenic region relative to the number that have 3′ ends within the gene body. We reasoned that levels of transcriptional readthrough might be altered by imatinib treatment compared with control and that the chemical inhibitor of CPC, JTE-607, could serve as a positive control for increased transcriptional readthrough of at least a subset of genes (*34*, *39*). Nascent RNA long-read libraries were prepared by isolating chromatin fractions (fig. S1A) from three biological replicates each for four different treatments: control (CTRL), 1-hour treatment with JTE-607 (JTE 1h), 1-hour treatment with imatinib (IM 1h), and 18-hour treatment with imatinib (IM 18h) (*40*). Read numbers, mapping statistics, and read lengths are available in tables S1 and S2. The read length distributions (fig. S1B), similar to previous nascent RNA long reads, allow us to analyze RNA processing early in gene transcription. We capture longer reads that might reflect transcriptional readthrough for approximately 11,000 genes per dataset (fig. S1C). Normalization indicates a similar read length distribution and number of representative genes across all datasets (fig. S1, D and E). Meta-analysis of the resulting read coverage across the gene body and extending 4 kb upstream of the TSS and downstream of the last PAS revealed that JTE-607 treatment led to transcriptional readthrough, as expected (Fig. 1C). Note that the decrease in transcriptional readthrough approaching +4 kb is in part determined by the length limitations of long-read sequencing (fig. S1, B, D, and F). Together, IM 1h and IM 18h treatments were characterized by higher levels of readthrough compared with CTRL and lower levels than JTE. These data raise the question of which genes are affected by imatinib.

To generate parameters from our data that would enable gene-specific analysis of nascent RNAs sequenced in our libraries, we defined readthrough reads as those that originate at either the TSS or within the gene but have 3′ ends ≥100 nucleotides (nt) downstream of the last annotated PAS (Fig. 1C). We defined reads with 3′ ends within the transcribed gene (i.e., between the TSS and the PAS) as “gene body” reads. This allowed us to derive a gene-specific metric, the readthrough index (RTI), which signifies the proportion of long reads that are not cleaved efficiently. Different treatment groups (Fig. 1D and fig. S2), had similar RTI distributions (fig. S2, A and B); however, the IM 1h dataset displayed significantly higher RTIs (Fig. 1D and fig. S2B). JTE 1h treatment was characterized by overall more genes (~1450 per million reads) with RTI > 0 compared with CTRL, IM 1h, and IM 18h, which each had 1000 to 1100 genes per million reads with readthrough (Fig. 1E). These data show that JTE inhibits the cleavage of some but not all genes, consistent with the findings of others (*34*, *39*), and that transcriptional readthrough is detected and quantifiable in nascent RNA long-read sequencing libraries.

To address whether imatinib treatment induces readthrough in different sets of genes, we analyzed unique and overlapping genes with RTI ≥ 0.2 (Fig. 1F). A total of 528 genes were common among CTRL and imatinib treatments. The sample with the most (819) unique genes with readthrough is IM 18h, while 449 genes in IM 1h and IM 18h were shared and not present in CTRL. To determine how the extent of readthrough varies between CTRL, IM 1h, and IM 18h, we created a heatmap with CTRL RTIs sorted from low to high by gene; corresponding RTI values for both imatinib treatments were aligned accordingly (Fig. 1G). The data show a range of altered intensities across the genes analyzed for IM 1h and IM 18h, with many similarities between the two time points. Thus, although IM 18h has the largest number of unique genes with RTI ≥ 0.2 (Fig. 1F), our data indicate a notable induction of transcriptional readthrough within 1 hour of imatinib treatment.

### Intron retention is associated with readthrough in all conditions

**Fig. 2.**
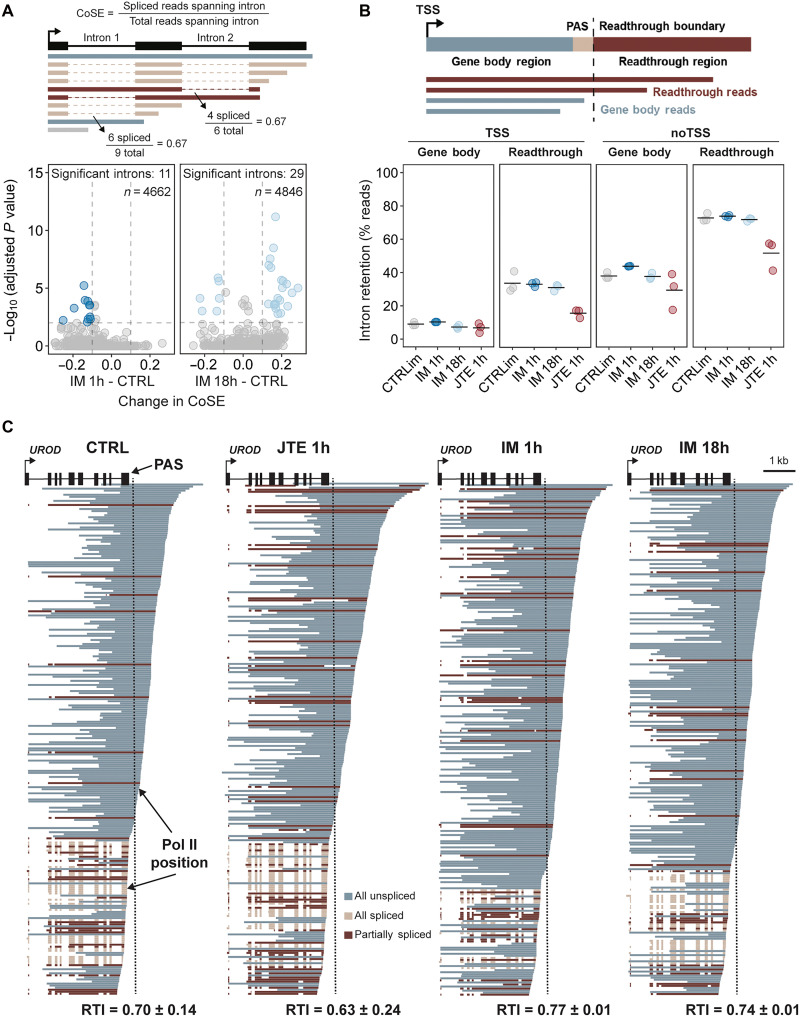
Transcriptional readthrough is associated with higher incidence of intron retention. K562 cells were untreated (CTRL) or treated with imatinib (IM) or JTE-607 (JTE) for 1 or 18 hours. (**A**) Analysis of CoSE based on long-read sequencing of nascent RNA. Top: Schematic explanation of CoSE metric. Bottom: Change in CoSE value for IM 1h or IM 18h relative to CTRL plotted for individual introns (*n* = 4662 total introns for 1-hour IM; 4846 for 18-hout IM analyses). Introns with significant changes in splicing efficiency (|Change in CoSE| ≥ 0.1 and adjusted *P* value < 0.05) are marked in blue. An adjusted *P* value was determined for each intron using chi-square tests for pairs of replicates and the Benjamini-Hochberg procedure. (**B**) Top: Schematic explanation of classification of long reads as gene body or “readthrough.” Bottom: Percentage of nascent RNA long reads with all introns retained (“all unspliced”). Reads were either filtered for those beginning within 100 bp of the transcription start site (TSS) or not filtered (noTSS). The black line represents the mean of three biological replicates. (**C**) Alignment of nascent RNA long reads to the gene uroporphyrinogen decarboxylase (*UROD*). Reads are colored according to their splicing status and downsampled to 250 reads in total. RTI values (means ± SEM) for *UROD* from three independent experiments are shown. The black dotted line represents the beginning of the readthrough region, and the 3′ end of each read corresponds to Pol II position.

Long reads also reveal splicing information that can be correlated with other features of each read within the same RNA molecule (*6*, *9*, *10*). Therefore, we sought to determine whether transcripts with transcriptional readthrough displayed elevated levels of intron retention and, if true, whether imatinib treatment altered this result. To do so, we quantified cotranscriptional splicing efficiency (CoSE) of CTRL, IM 1h, and IM 18h, by aligning our long reads to gene annotations (Fig. 2A, top). Relatively few introns showed substantial changes in CoSE in either the positive (more splicing) or negative (intron retention) direction after 1- or 18-hour IM treatment (Fig. 2A, bottom) or after JTE treatment (fig. S3A). Only ~10% of introns were retained in full-length reads that begin at the TSS and have 3′ ends within the gene body, consistent with efficient cotranscriptional splicing and unaffected by IM 1h or IM 18h (Fig. 2B). As expected, more introns (~35%) were retained in readthrough reads, and this was similarly unaffected by imatinib. Notably, JTE treatment reduced intron retention in the fraction of readthrough genes that could be caused by its sequence-dependent impact on splicing (*34*, *39*). Because of read length limitations, focusing on full-length reads decreases the number of intron-containing readthrough reads that we can analyze. Therefore, we separately analyzed readthrough reads that did not start at the TSS and found ~40 and ~75% retained introns (RIs) in gene body and readthrough categories, respectively. Again, neither parameter was significantly affected by imatinib (Fig. 2B). However, 1-hour treatment with imatinib significantly increased intron retention detected by CoSE in transcripts with lower RTI (fig. S3B). A partial correlation between transcriptional readthrough and gene-specific CoSE was reinforced by global analysis, reflecting the increased intron retention present in readthrough transcripts (fig. S3, B and C). We conclude that readthrough transcripts are correlated with intron retention in human K562 cells and that imatinib does not increase intron retention levels independently of transcriptional readthrough.

In a previous study, we found that transcripts from the β-globin gene and other erythroid genes displayed “all-or-none” RNA processing, in which half of the transcripts were efficiently spliced and cleaved at the 3′ end (*6*). The other half contained all of their introns and displayed transcriptional readthrough. We searched for genes displaying all-or-none processing (RTI ≥ 0.2 and CoSE ≤ 0.7) and found 44 genes exhibiting this behavior, one of which is uroporphyrinogen decarboxylase (*UROD*) (Fig. 2C). Alignment of subsampled *UROD* transcripts shows partially spliced, all spliced, and all unspliced transcripts displayed in the order of Pol II elongation from TSS to PAS. As expected, the majority of readthrough transcripts are unspliced, and both IM 1h and IM 18h treatments increase the RTI. The *UROD* gene encoded decarboxylase is crucial for heme production. Gene Ontology term analysis for the genes with RTI ≥ 0.2 in IM 1h and IM 18h highlights metal ion binding and enzymatic activities characteristic of erythropoiesis (fig. S4, A and B), suggesting that imatinib is able to direct K562 cells into the erythroid lineage on shorter timescales than previously appreciated (*41*).

### Erythroid differentiation accompanies transcriptional readthrough upon imatinib treatment

**Fig. 3.**
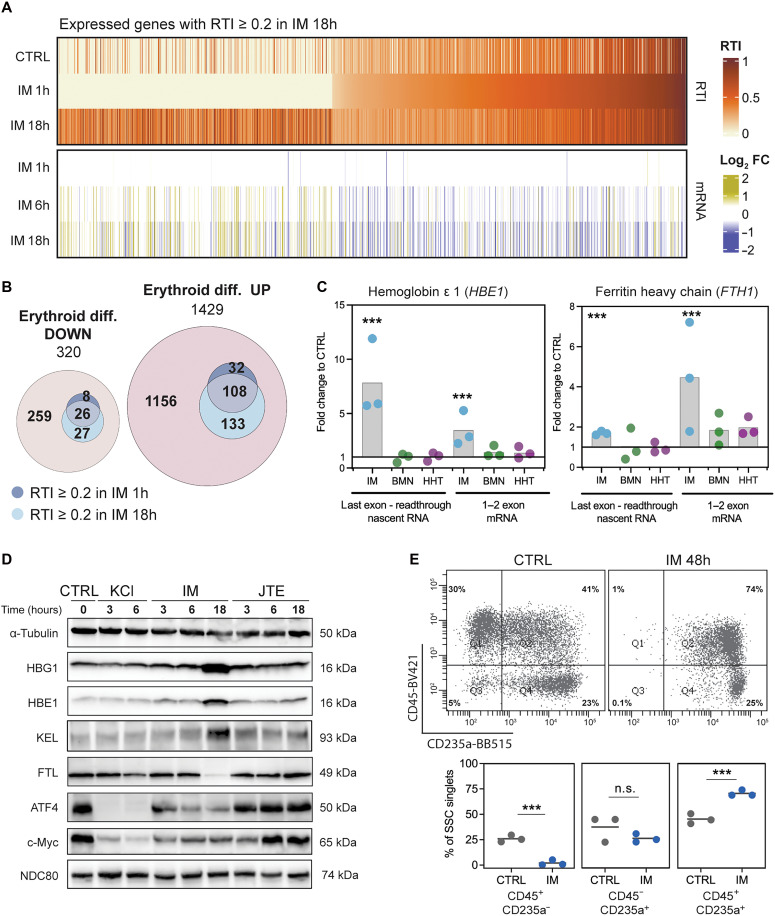
Imatinib-induced transcriptional readthrough is present in genes associated with erythropoiesis. (**A**) Changes in RTI and mRNA levels upon IM treatment for genes with RTI ≥ 0.2 in IM 18h (*n* = 1818), ordered by RTI in IM 1h. Log_2_FC, log_2_ of fold change relative to CTRL. (**B**) Prevalence of genes with RTI ≥ 0.2 after IM 1h or IM 18h overlapping with genes that are up- or down-regulated upon progenitor differentiation into poly- and ortho-erythroid cells (Erythroid diff. UP or DOWN, respectively). Values represent numbers of genes. (**C**) Changes in transcriptional readthrough and mRNA levels upon 18-hour treatment with imatinib (IM), talazoparib (BMN), or homoharringtonine (HHT) quantified for hemoglobin e (*HBE1*) and ferritin heavy chain (*FTH1*) using RT-qPCR. Significance based on two-tailed Student’s *t* test (***P < 0.005). (**D**) Western blot detection of proteins encoded by selected up- and down-regulated genes important in erythroid differentiation and cell cycle regulation. Equal amounts of protein from whole-cell lysates were loaded in each lane; α-tubulin for reference. (**E**) Erythroid differentiation markers analyzed by flow cytometry in CTRL and cells treated with IM for 48 hours (IM 48 h). Antibodies conjugated to fluorophores BV421 and BB515 were used for detection of CD45 and CD235a proteins, respectively. Top: Representative plot showing percentage of cells positive for each CD marker. Bottom: Black line represents mean value of three replicates (points). Significance based on two-tailed Student’s *t* test (***P < 0.005). n.s., not significant.

To determine the cellular significance of these early gene expression changes in response to imatinib, we generated datasets from short-read polyA^+^ RNA-seq libraries to corroborate and extend our findings (table S1). To ask whether the genes with high RTI (see Fig. 1G) belonged to previously or newly expressed genes, we analyzed 1818 genes with RTI ≥ 0.2 in IM 18h samples; RTI values per gene were sorted according to the IM 1h dataset to visualize how readthrough and gene expression change over time (Fig. 3A, top). The same 1818 genes were sorted in the same order and then expressed in terms of fold change in mRNA level compared to control, showing that only minor changes in mRNA levels were detected at 1 hour (Fig. 3A, bottom). Notably, the data at 6 and 18 hours clearly indicate that 78% of the genes with high RTI values at 1 hour are down-regulated over the time period. In contrast, the genes with low RTI at 1 hour are mostly up-regulated and increase in RTI by 18 hours, accounting for the increased numbers of readthrough genes by the later time point. By comparison, only 9 and 5%, respectively, of the 1491 genes with readthrough upon 1-hour JTE treatment (133 genes at 6 hours and 73 genes at 18 hours) were down-regulated; ≤1% were up-regulated (18 and 8 genes at 6 and 18 hours, respectively; fig. S5). We conclude that genes with substantial readthrough at 1 hour of imatinib treatment are on a trajectory of decreasing gene expression, which could be caused by either repressed transcription and/or a relative decrease in transcript stability owing to the failure to cleave at the PAS.

Analysis of differential gene expression revealed an increase in some erythroid genes, such as hemoglobins, even after 1 hour of imatinib treatment (fig. S6A). Thus, we checked whether genes with transcriptional readthrough upon IM 1h or IM 18h in K562 cells belong to the constellation of genes (erythroid or nonerythroid) that are up- or down-regulated during normal erythropoiesis. For reference, we used a dataset from human CD34^+^ hematopoietic stem cell progenitors undergoing differentiation into poly- and ortho-erythroid cells (*42*). Three hundred thirty-four of the identified genes with RTI ≥ 0.2 (16%) overlapped with the normal erythropoiesis dataset, increasing in number from 1 to 18 hours of imatinib (Fig. 3B). Mean RTI for these overlapping genes reveals a trend to higher RTI in IM 1h (fig. S6B). We used RT-qPCR to validate two examples—hemoglobin ε (*HBE1*) and ferritin heavy chain (*FTH1*)—that displayed increased transcriptional readthrough and mRNA levels in IM 18h (Fig. 3C). Neither of two other chemotherapeutics with distinct mechanisms of action—talazoparib (BMN) or homoharringtonine (HHT)—induced transcriptional readthrough, indicating the specificity of imatinib’s effects on 3′-end cleavage, at least for these two genes. To further validate the specific, early induction of erythropoietic genes by imatinib, we performed Western blotting and detected elevated globins γ and ε as well as Kell endopeptidase (*KEL*) in IM 18h samples (Fig. 3D). Last, flow cytometry analysis of K562 cells treated with imatinib for 48 hours confirmed their differentiation to CD45^+^ CD235a^+^ erythroblasts (Fig. 3E), validating and extending previous findings (*41*).

### Imatinib leads to alternative mRNA isoforms with and without transcriptional readthrough

**Fig. 4.**
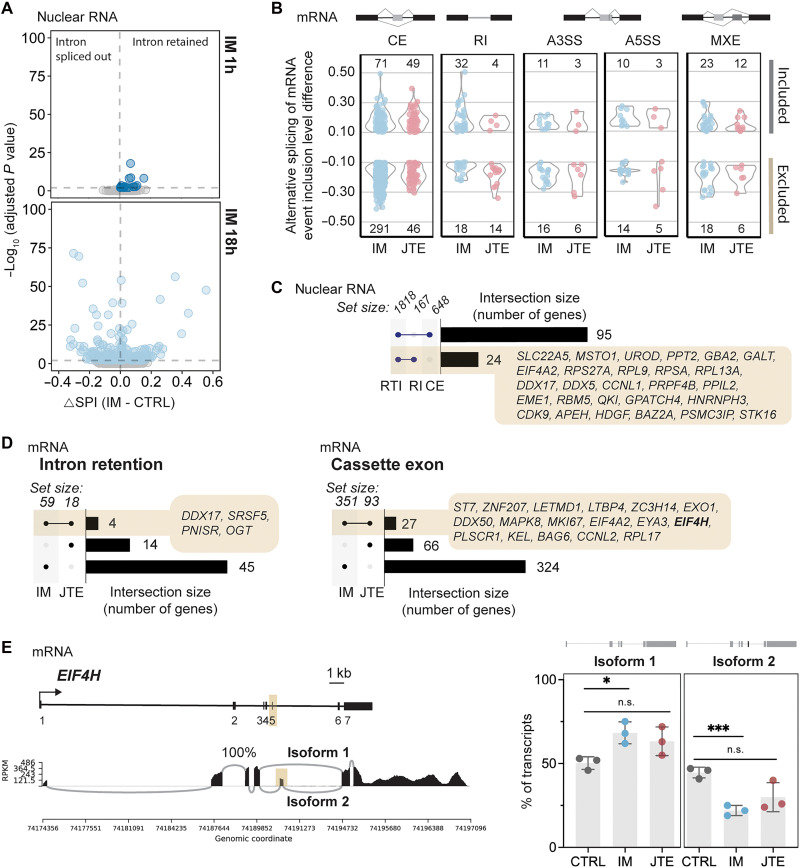
Widespread alternative splicing in response to imatinib correlates with readthrough in some cases. (**A**) Change in SPI values from short-read sequencing of mRNA in IM 1h (top) or 18 h (bottom) relative to CTRL. Introns (*n* = ~35,300) detected by at least 500 reads and with significant changes in SPI (adjusted *P* value < 0.05) are marked in blue (20 in IM 1h and 193 in IM 18h). Adjusted *P* values were determined using chi-square tests for pairs of replicates and the Benjamini-Hochberg procedure. (**B**) Significant changes in alternative splicing in cells treated with JTE or IM 18h. A3SS or A5SS, alternative 3′ or 5′ splice site; MXE, mutually exclusive events. Number of significant events [false discovery rate (FDR) < 0.05] detected by at least 20 reads and with absolute inclusion level difference to CTRL > 0.1. (**C**) Comparison of genes with RTI ≥ 0.2 (RTI) and genes with significant changes in RI and CE at the level of nuclear RNA in IM 18h. Only single intersections with RTI are presented. (**D**) Comparison of genes with significant changes in RI (left) or CE (right) in IM or JTE 18h. (**E**) Sashimi plot for *EIF4H* (left) and quantification of isoform usage (right) in CTRL versus JTE or IM 18h. Isoforms 1 and 2 represent the skipping and inclusion, respectively, of exon 5 (beige highlight). Percentage of transcripts belonging to each isoform in three replicates (means ± SEM). Significance based on two-tailed Student’s *t* test (*P < 0.05; ***P < 0.001).

It is well known that alternative splicing and intron retention are regulatory features of erythropoiesis (*42*–*44*). Since readthrough and intron retention are early responses to imatinib treatment, we wondered whether and when alternative splice isoforms might be detected. To rigorously address this, we generated short-read sequencing datasets from total nuclear RNA (table S3); our rationale was (i) changes in splicing will first be detected in the nucleus before achieving steady-state mRNA representation in the cytoplasm and (ii) intron retention events are better detected in the nuclear fraction. Using splicing per intron (SPI) as a statistically powerful metric, we detected a few intron retention events in IM 1h and an increase in positive and negative splicing changes at 18 hours (Fig. 4A), in agreement with the long-read nascent RNA-seq results (see Fig. 2A). Reduced mRNA levels for proteins involved in the regulation of splicing (fig. S7A), including serine/arginine-rich splicing factor 1 (SRSF1) as previously described (*45*), were detected after imatinib treatment. We confirmed down-regulation Gemin5 and serine-arginine protein kinase 1 (SRPK1) levels by immunoblotting (fig. S7B). We observed reduced level of WD repeat-containing protein 43 (WDR43), representative of a pool of imatinib–down-regulated proteins involved in ribosome biogenesis (fig. S7B), that was preceded by an increase in RTI score upon 1 hour of imatinib treatment (from 0.37 to 0.49). In contrast, increased *RBM5* mRNA was not reflected at the protein level, presumably because of intron retention and nuclear degradation of these transcripts (fig. S7C). We conclude that the mRNA and, in some cases, protein levels of stimulatory RNA processing factors decrease upon imatinib treatment.

Imatinib, by affecting the expression of factors involved in splicing regulation, should affect alternative splicing and thereby influence cell response to treatment. SR protein kinase 1 modulates the activity of splicing factors with serine/arginine domain, and its down-regulation offers a direct explanation of the imatinib link to the stress-induced changes in alternative splicing (*46*). Suppression of SRPK1 by imatinib induces cell death in K562 cells (*47*). Also, altered level of Gemin5 was found to modulate alternative splicing of a spectrum of transcripts (*48*), and its up-regulation causes changes primarily in exon skipping (*49*). Analysis of our mRNA sequencing (mRNA-seq) dataset was conducted, comparing CTRL to IM and JTE 18h using the replicate multivariate analysis of transcript splicing (rMATS). This revealed ~500 alternative splicing events in IM 18h, greater than 10 times the number of changes in IM 1h (fig. S8A), including a large number of cassette exon (CE) events, both inclusion and exclusion (Fig. 4B). As a control for possible direct or indirect effects of transcriptional readthrough on alternative splicing, a dataset was collected for JTE 1h and JTE 18h; approximately 40 and 150 significant alternative splicing changes were detected, respectively (fig. S8A). By contrast, another stress condition associated with transcriptional readthrough—hyperosmotic shock—led to almost 400 alternative splicing events, including many CEs and RIs, after only 1 hour of 110 mM KCl treatment (fig. S8, A and B). We conclude that direct inhibition of 3′-end cleavage by JTE itself leads to modest but significant numbers of alternative splicing events, suggesting that some of the alternative mRNA isoforms induced by IM could be due to readthrough. However, in addition, alternative splicing is a robust and specific response to imatinib treatment that differs from JTE treatment and hyperosmotic shock.

To determine whether alternative mRNA isoforms induced by imatinib are linked to transcriptional readthrough, we performed two types of analyses. First, we asked whether RIs and CEs detected in nuclear RNA by alternative splicing analysis with rMATS were correlated with genes with RTI ≥ 0.2. Only 95 of 648 genes had both changes in CE inclusion and high RTI, and only 24 of 167 genes had both RI and high RTI (Fig. 4C). This set of RI genes includes erythropoietic genes, like *UROD*, and other genes that will be highlighted below. Intron retention in *DDX17* mRNA is displayed in detail and validated by RT-PCR in fig. S5 (C and D). Increased intron retention in *EIF4A2* is likely driven by the presence of five small nucleolar RNAs (snoRNAs) in different introns (*50*). Second, we compared sets of alternative mRNA isoforms detected in IM and JTE 18h (see Fig. 4B) to determine the extent of gene overlap. For intron retention (Fig. 4D, left), we find that only four genes are common between IM and JTE, while 45 intron-retained isoforms are unique to IM. Similarly, 324 cases of CE usage were not present in the JTE dataset (Fig. 4D, right), in agreement with data discussed above that transcriptional readthrough does not directly cause intron retention in these genes.

To explore a functionally important example of alternative splicing induced by JTE (of 93 cases of CE detected), we selected the mRNA encoding the eukaryotic translation initiation factor 4H (*EIF4H*) harboring a CE (exon 5) that defines two different protein isoforms (Fig. 4E, left). Inclusion of short peptide sequence in eIF4H longer isoform induces conformational change that could potentiate a protein-protein interaction with eIF4A helicase thereby increasing translation of selected transcripts and contributing to cancerogenesis (*51*–*53*). Quantification of splicing events (Fig. 4E, right) and RT-PCR validation (fig. S8E) revealed that both IM and JTE significantly increase the proportion of isoform 1 and decrease the proportion of isoform 2, containing exon 5. Because 27 of the CE events induced by JTE overlap with those induced by IM, including *EIF4H*, it is therefore possible that these alternative splicing events are directly linked to transcriptional readthrough in both cases. A summary of the observed differential splicing changes attributable to the erythroid gene expression program reveals extensive alternative splicing regulation of numerous up- and down-regulated genes (fig. S8F).

**Fig. 5.**
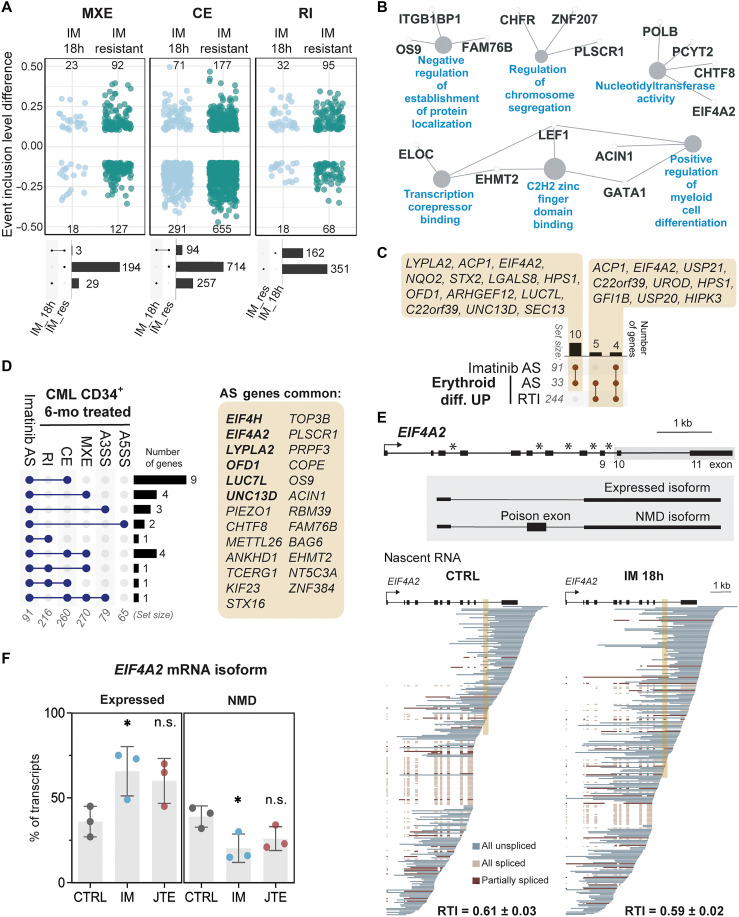
Early changes in alternative splicing control response to imatinib in a longer time course. (**A**) Comparison of IM-resistant K562 cells (GSE267522) and K562 cells treated with IM for 18 h (IM 18h). Top: Significant changes (FDR < 0.05) in alternative splicing (AS), presented events detected by at least 20 reads. MXE, mutually exclusive events. Number of significant events with absolute inclusion level difference to control cells >0.1. Bottom: Upset plots comparing events induced by each condition. (**B**) Enriched Gene Ontology Biological Process terms (blue) for genes that are differentially spliced in both IM-resistant and IM 18h–treated cells [MXE and CE from (A), bottom]. (**C**) Comparison of genes that are up-regulated upon erythroid differentiation (Erythroid diff. UP) and have RTI values ≥0.2 (RTI) or have significant AS changes upon IM 18h treatment (AS) with genes that have significant changes in CE or MXE for both IM-resistant cells and IM 18h (Imatinib AS). (**D**) Comparison of significant AS changes for both IM-resistant and IM 18h K562 cells (Imatinib AS) and changes for CD34^+^-enriched cells from a patient with CML at diagnosis and after 6 months of IM treatment. (**E**) Top: Architecture of eukaryotic translation initiation factor 4A2 (*EIF4A2*). SnoRNAs indicated by black boxes with stars. Bottom: Downsampled nascent RNA long reads colored according to splicing status, with shaded box over poison exon. RTI values (means ± SEM) for *EIF4A2* from three independent experiments are shown. (**F**) Quantification of isoform usage for *EIF4A2*. The NMD and expressed isoforms represent inclusion and skipping, respectively, of the poison exon. Percentage of transcripts belonging to each isoform in three replicates (means ± SEM). Significance based on two-tailed Student’s *t* test (*P < 0.05).

Given our early detection of imatinib-induced transitions to cell fates (e.g., erythropoiesis), we considered the possibility that some of the observed alternative splicing could be related to the development of imatinib resistance. We analyzed an mRNA-seq dataset from K562 cells treated with a progressively increasing concentration of imatinib over a period of 10 months (*54*). These cells are imatinib resistant and display a large number of alternative splicing changes: 97 mutually exclusive exons, 808 CEs, and 162 RIs (Fig. 5A). Although none of the identities of RIs overlap with our IM 18h dataset, 94 cassette and 3 mutually exclusive exons splicing events did overlap. Thus, alternative splicing may contribute to early steps toward long-term imatinib resistance, provided at the cellular adaptation level and independent of genomic mutation–driven resistance. This includes *NFE2L1*, which encodes an endoplasmic reticulum–localized protein that translocates to the nucleus upon stress, where it is involved in the regulation of erythroid differentiation as a transcription factor (*NRF1*) [for review, see (*55*)]. Results of RT-PCR across the exon validate the change in isoforms (fig. S8, G and H). Gene Ontology analysis for these overlapping alternatively spliced genes revealed many changes consistent with progression to other cell fates, such as transcription factors that promote myeloid differentiation and factors that control proper chromosome segregation (Fig. 5B). Many of the overlapping genes are related to erythroid differentiation, showing that development of imatinib resistance involves at least a partial shift to that gene expression program (Fig. 5C). To determine whether these changes are comparable to a patient treated with imatinib, we performed mRNA-seq on CD34^+^ cells isolated at diagnosis and after 6 months of treatment (Fig. 5D). Comparing the different categories of numerous alternative splicing changes present in the patient samples after imatinib treatment, 26 were common with our IM 18h sample, of which 6 genes belonged to the erythroid gene expression program. One example is the translation initiation factor *EIF4A2*, which contains a “poison cassette” exon that harbors premature stop codons, triggering nonsense-mediated decay (*56*). Elevated inclusion of this exon is visible in the nascent RNA long-read sequencing dataset and validated by RT-PCR (Fig. 5E and fig. S8I). The relative abundances of the nonsense-mediated mRNA decay (NMD) and expressed isoforms are negatively correlated (Fig. 5F), indicating that potentially more EIF4A2 protein is expressed in response to imatinib treatment.

### Imatinib modulates the formation of chimeric mRNAs

**Fig. 6.**
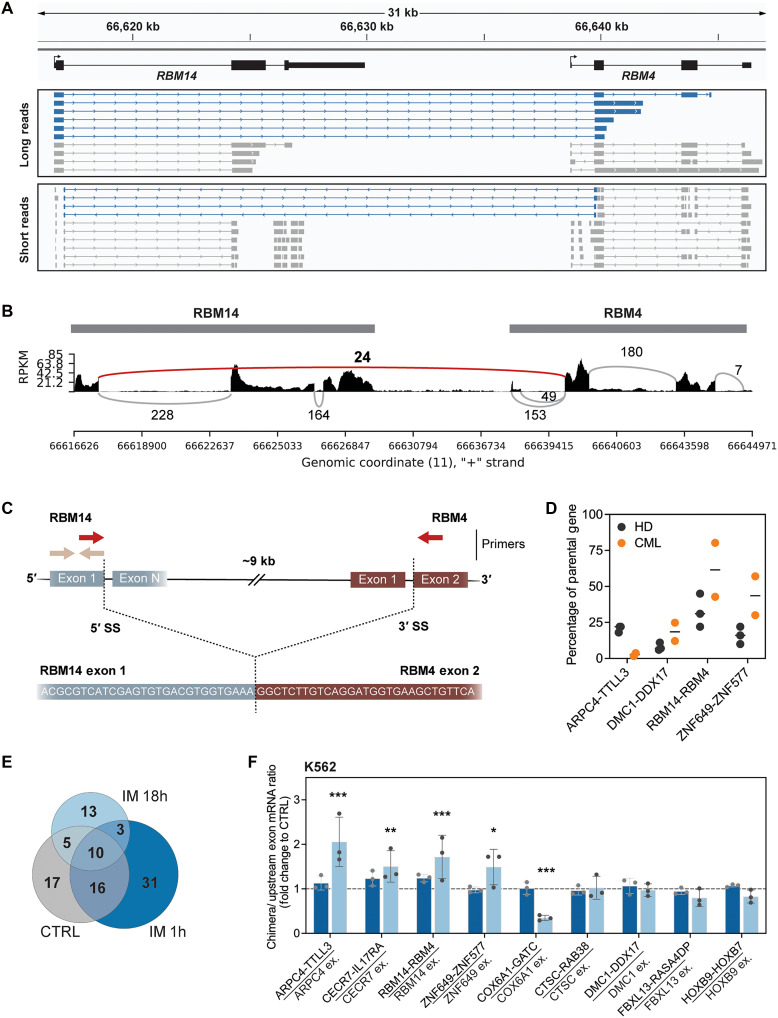
Imatinib-induced formation of readthrough chimeras. (**A**) Examples of reads aligned to *RBM14* and *RBM4* from long- and short-read sequencing of IM-treated cells. Chimeric reads marked in blue. (**B**) Sashimi plot for *RBM14* and *RBM4* (chromosome 11: 66616624 to 66644972) based on a single representative CTRL replicate. Only splicing events detected more than five times are shown. Black represents read coverage density in RPKM, gray lines represent splicing events within gene bodies, and the red line represents chimeric splicing. (**C**) Scheme of primer design for real-time PCR semiquantification (qPCR) of chimera expression. (**D**) Expression of selected chimeras [red primers in (C)] in CD34^+^-enriched cells from three healthy donors (HD) and two patients with CML (CML), normalized to spike-in controls and presented relative to the expression level of the last exon in the upstream gene [beige primers in (C)]. (**E**) Comparison of readthrough chimeras identified by SOAPfuse analysis of mRNA-seq for CTRL or IM-treated K562 cells. (**F**) Changes in chimera transcript expression normalized to spike-in control referenced to the last exon in the upstream gene and expressed as fold change upon IM treatment. Level in CTRL as 1.0, marked with the dashed line. Significance based on two-way ANOVA test (*P < 0.05; **P < 0.005; ***P < 0.001).

Transcriptional readthrough can lead to the formation of RNA chimeras and even produce fusion proteins from those spliced mRNA products (*20*, *21*). We used bioinformatic tools to search for these “readthrough chimeras” from K562 total and nuclear RNA-seq, as well as CD34^+^ cells [including publicly available datasets; (*57*)], and sought to validate them with our long-read data. The dataset with the most chimeric reads was imatinib-resistant K562 cells and CD34^+^ from patients with CML (fig. S9, A and B). A notable number of chimeras were also detected by short and long reads in K562 cells at 18 hours, including the *RBM14-RBM4* chimera in which the 5′ SS at the end of *RBM14* exon 1 is spliced to the 3′ splice site of the *RBM4* exon 2 (Fig. 6, A and B, and fig. S9, A and B). These chimeras correlated with elevated RTI values from the upstream-most gene (fig. S9C), and we see that transcription of the downstream gene may increase and/or be coordinated with the upstream gene over a time course of imatinib treatment (fig. S9, D and E).

To quantify expression and the relative abundance of the single RNA and chimeric RNA isoforms in physiologically relevant cells, we prepared total RNA from CD34^+^ cells from three healthy and two CML patient donors. We designed specific primers to amplify either the first exon (or a region of the upstream parental gene) to which the abundance of the chimera—detected with primers across the exon-exon junction—would be normalized (Fig. 6C and table S4), using RT-qPCR. Analysis of four chimeras showed significant expression of *RBM14-RBM4* and *ZNF649-ZNF577* chimeric mRNAs in healthy cells that increased substantially in CD34^+^ cells from patients with CML. In contrast, variable amounts of *ARPC4-TTL3* and *DMC1-DDX17* chimeras were detected in these primary cells. Analysis of our K562 datasets revealed that chimeras are expressed in CTRL (48), IM 1h (60), and IM 18h (31) with significant overlaps in detection (Fig. 6E). Quantification of chimeras by RT-qPCR, using the above method, revealed that some—namely, *ARPC4-TTL3*, *CECR7-IL17RA*, *RBM14-RBM4*, and *ZNF649-ZNF577*—increase significantly by 18 hours of imatinib treatment with little or no increase at 1 hour (Fig. 6F and fig. S10A), placing them in the category of alternative splicing changes seen at the later time point. In contrast, *COX6A1-GATC* is significantly down-regulated, while other chimeras do not change. Treatment of healthy and CML donor cells with IM 18h did not markedly change absolute levels of chimeras relative to parental genes (fig. S10B). However, *ARPC4-TTL3* and *RBM14-RBM4* did increase by up to threefold relative to CTRL when healthy donor cells were treated for 18 hours and *DMC1-DDX17* increased when CML donors received 1 hour of imatinib, suggesting the potential for physiological relevance for these cells. Therefore, we conclude that expression of readthrough chimeras is part of a response to imatinib treatment, enabled by both transcriptional readthrough and alternative splicing changes.

## DISCUSSION

**Fig. 7.**
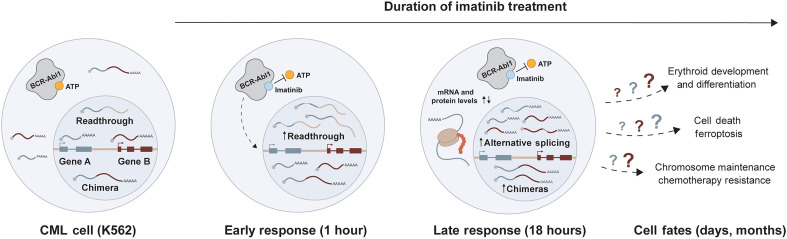
Working model: A cascade of molecular mechanisms triggered by imatinib treatment of CML cells. Transcriptional readthrough and intron retention are the first major changes detected at 1 hour. At 18 hours, increasing intron retention, alternative splicing, and readthrough chimera formation may be associated with induced cell fates, such as erythropoiesis, and the development of resistance to therapy.

Here, we have used precision RNA-seq methods to analyze the earliest changes in gene expression that take place in K562 cells and cells of patients with CML upon chemotherapy. Overall, initial responses to imatinib, which directly targets the activity of the *BCR-ABL1* oncogene, are multifaceted within the first 24 hours. We depict the relative timing and importance of this cascade of gene expression changes as a series of steps and cellular options (Fig. 7). First, long-read sequencing of nascent RNA identified immediate activation of transcriptional readthrough, a blockage or delay in the cleavage of nascent RNA 3′ ends (*11*, *12*). To our knowledge, this is the first identifiable change in gene expression caused by imatinib, which is known to cause cell death, erythroid differentiation, and chemotherapy resistance at much later time points. Second, RNA signatures of longer-term imatinib treatment, such as elevation in erythroid gene expression, were detected as early as 1 hour. Previous studies identified development of imatinib resistance in K562 cells (*41*) involving protection from cell death by ferroptosis (*58*) as a simultaneous occurrence with erythropoiesis. These unexpectedly early changes in transcriptional readthrough and gene expression may have prognostic value, because higher expression of the CD235a erythroid marker is consistent with high efficacy of imatinib treatment and the acquisition of major molecular remission (*59*). Third, changes in alternative splicing at 18 hours of imatinib treatment were also detected in imatinib-resistant K562 cells (10 months) as well as samples from a patient with CML collected at diagnosis and after imatinib treatment (6 months), further suggesting potential early roles in the development of chemotherapy resistance. Fourth, we found chimeric RNAs that were induced by imatinib and elevated in patients with CML versus healthy donors, emphasizing the outcome of two coordinated events: (i) repression of splicing in the upstream gene and (ii) transcriptional readthrough into the downstream gene; these two events enable the elevated expression of chimeric RNAs and potential previously unknown protein products in CML cells. We discuss these features in more depth below.

The identification of readthrough as an early event was facilitated by its quantification from long-read sequencing of nascent RNA, revealing the proportion of transcripts that are uncleaved at the last annotated polyA cleavage site. The long reads establish the molecular integrity of each RNA molecule, ruling out the possibility that transcripts initiating in downstream intergenic space would be counted. This differs from previous determinations of transcriptional readthrough, which quantify short-read density downstream of gene ends (*15*, *16*, *18*, *32*, *60*). The mechanisms that lead to early readthrough upon imatinib treatment are not currently known and require further investigation. We show that the induction of readthrough is specific to imatinib as a therapeutic and not seen with homoharringtonin or talazoparib. Imatinib’s triggering mechanism is also unlikely to be a direct inhibition of the CPC, because JTE—a drug that does directly suppress cleavage—generates a larger number of readthrough genes that is largely nonoverlapping with those triggered by imatinib. Intron retention, which is associated with transcriptional readthrough, could be a cause or an effect of transcriptional readthrough; indeed, prior studies in budding yeast showed that intron retention is caused by transcriptional readthrough and vice versa (*10*, *61*).

Our time-resolved study provides a rigorous metric—the RTI—that enables recognizing transcriptional readthrough as an underlying mechanism related to splicing regulation and erythropoiesis. For example, transcriptional readthrough was associated with inefficient splicing in some of the induced erythroid genes (e.g., *UROD*, *HBE1*, and *FTH1*), although these genes are overall strongly expressed. We showed that splicing factor mRNA and protein levels encoding splicing factors decrease following the early detection of readthrough in these genes. Thus, mechanistically, readthrough may lead to down-regulation of their gene of origin and ultimately determine the fraction of cells able to attain the erythroid fate. One demonstrated mechanism of down-regulation includes intron retention or inclusion of poison exons, both of which reduce mRNA levels by activating nonsense-mediated decay (*56*), consistent with our data. Transcriptional readthrough correlated with other forms of alternative splicing; *DDX17* and *EIF4H* underwent alternative splicing upon both JTE and imatinib treatments, suggesting that readthrough alone—as opposed to a cellular stress response activated by imatinib treatment—drives alternative splicing in some cases. Because JTE does not induce erythropoiesis, these events have to be seen as differing from the red cell differentiation program activated by imatinib. Long-term responses to imatinib included changes in CE inclusion and intron retention, consistent with previous findings (*62*). Considering the potential cascade of molecular events over time (Fig. 7), changes in the expression of splicing factors could lead to downstream changes in mRNA isoform levels (*63*). Further studies will be needed to identify the specific relationships between changes in individual splicing factors and mRNA isoforms expressed.

From a pool of alternative isoform changes induced by IM 18h, we identified encoded factors that define erythroid differentiation, such as *GATA1* and *NFE2L1/NRF1* (transcription factors) and *UROD* (a cytoplasmic enzyme involved in the heme biosynthesis pathway). In K562 cells, erythroid differentiation manifested by increased expression of hemoglobin can be induced by hemin (*64*) and increases cell survival upon imatinib (*65*–*67*). Increased level of TCF11/NRF1 induces expression of proteasome, thereby maintaining proteostasis and playing a cytoprotective role (*68*, *69*) in CML cells with increased activation of the unfolded protein response (*26*, *27*). Imatinib caused increased expression of the longer *NFE2L1* isoform, also known as *TCF11*. The Nhe4l domain, encoded by the alternative exon, plays a role in NRF1/TCF11 dimerization with other transcription factor MAFG (*70*) and alters the spectrum of genes regulated by NRF1/TCF11 (*62*). Increased expression of *UROD* was observed in *MYCN*-expressing acute lymphoblastic leukemia, and imbalanced synthesis of porphyrins determined cancer cell survival (*71*). As postulated recently (*61*), transcriptional readthrough might facilitate alternative splicing decisions in such cases. We show that alternative mRNA isoforms expressed in imatinib-resistant K562 cells (10 months) occurred much earlier, at 18 hours of treatment, demonstrating these RNAs as potential molecular signatures of chemotherapy resistance. We identified reduced poison exon inclusion in *EIF4A2* transcripts already within 18 hours of imatinib treatment. Inclusion of the poison CE between exons 10 and 11 produced a truncated protein that was rapidly degraded in differentiating cardiomyoblasts (*72*). Therefore, we infer that reduced inclusion of the *EIF4A2* poison exon would greatly increase protein expression. Given that the promotion of cell growth and proliferation by Bcr-Abl1 involve stimulation of translation (*56*), this could serve as a rescue mechanism that promotes the progression of cancer despite treatment. This agrees with an earlier observation that alternative splicing of *EIF4A2* was essential for increased protein levels in the development of AML (*73*).

Effects of transcriptional readthrough and alternative splicing encompass the generation of chimeric transcripts (*10*, *16*, *20*, *21*). Chimeric transcripts may be hallmarks of RNA processing in oncogenesis and response to therapy (*74*). The mechanism for generating RNA chimeras between two genes in tandem must not only involve transcription from the upstream gene into the downstream gene, but, in addition, intron retention in the upstream gene is required to provide a 5′ splice site in the upstream gene that can be spliced to a 3′ splice site in the downstream gene (*20*). Thus, the modification of alternative splicing patterns accompanying transcriptional readthrough upon imatinib would drive the generation of new and/or elevated chimeric transcripts. Reduced levels of SRSF1, SRPK1, and/or Gemin5 could affect the readthrough chimera cis-splicing. By far, SRRM1 and SF3B1 were identified as possible regulators of the chimeric transcript cis-splicing (*75*). Their activity is modulated by SRPK1-mediated phosphorylation of SRRM1 or SRSF1. If translated, the resulting chimeric proteins might display gain or loss of function of the original proteins and have different stability, cellular localization, or a modified spectrum of interactors. This has been observed in the case of *RBM14-RBM4* (*76*). Apart from protein-protein fusions, we also observed long noncoding RNA (lncRNA)–protein chimeric transcripts, like lncRNA *CECR7-IL17RA*. Stimulation of *IL17RA* cell surface receptor plays a prosurvival role in leukemia, and targeting of interleukin- 17A cytokine increases imatinib’s effectiveness in Bcr-Abl1–positive acute lymphoblastic leukemia (*77*). In addition, so-called “ripples” in Pol II transcriptional activity in intergenic regions are associated with chromatin changes that could lead to altered protein-coding gene expression (*78*). Epigenetic changes accompanying erythroid differentiation (*79*) could perhaps support CML resistance (*80*) by broadly adjusting gene expression.

Together, the early modifications in RNA processing that we observed upon imatinib treatment—including transcriptional readthrough, intron retention, alternative splicing, and readthrough chimera formation—could be exploited as a potential vulnerability after further study. Fusion proteins synthesized from chimeric transcripts can serve as diagnostic markers or neoepitopes for targeted T cell–mediated therapy (*81*) or anticancer vaccines (*82*). Similarly, peptides originating from nonsense-mediated decay of translated transcripts with RIs were found to be presented by major histocompatibility complex I (*83*). This underscores the importance of the detection of RNA processing changes as early as 1 hour and progressing through an early time frame of gene expression changes in response to imatinib. Identified changes in RNA processing and detection of isoforms can deliver robust hallmarks of cell responses to therapeutics, like induction of cell differentiation, possibly allowing for early detection of arising therapy resistance.

## MATERIALS AND METHODS

### Cell treatments and fractionation

K562 cells were cultured in Iscove’s modified Dulbecco’s medium supplemented with 10% fetal bovine serum, penicillin (100 U/ml), and streptomycin (100 μg/ml). Cells were treated with 110 mM KCl, 5 μM JTE, or 1 μM imatinib for 45 min, 1 hour, 3 hours, 6 hours, or 18 hours, as indicated in the figure legends. Each experiment was performed in triplicate to obtain three biological replicates. After washing in cold phosphate-buffered saline (PBS), 8 × 10^7^ cells were subjected to fractionation to obtain chromatin-associated nascent RNA, as described (*6*, *40*). The only modification was an increase in the number of times the pellet was washed with PBS between fractionation steps from one wash to three washes. Fractionation was performed in four technical replicates, with 2 × 10^7^ cells for each replicate. One of the replicates was used to assay the isolated cytoplasmic, nuclear, and chromatin fractions by Western blotting. To this end, cold PBS was added to each of the cell and nucleus lysis supernatants to achieve the same final volume. The corresponding volume of PBS was also added to the chromatin pellet. Then, the samples were sonicated on ice at 15% amplitude for 20 s and centrifuged for 10 min at 20,000*g*, 4°C. Aliquots of the same volume from each supernatant were incubated with a NuPAGE sample buffer (Thermo Fisher Scientific, no. NP0007) at 95°C for 5 min before subjecting them to NuPAGE electrophoresis in a 4 to 12% Bis-Tris gel (Thermo Fisher Scientific, no. NP0322PK2) and Mops-SDS buffer (Thermo Fisher Scientific, no. NP0001). Following transfer, the nitrocellulose membranes were incubated at 4°C overnight with primary antibodies against glyceraldehyde phosphate dehydrogenase (Santa Cruz Biotechnology, no. sc-25778), Pol II (4H8) (Santa Cruz Biotechnology, no. sc-47701), histone H3 (Novus, no. NB500-171), and α-tubulin (Sigma-Aldrich, no. T6074).

Primary CD34^+^ cells were enriched from samples of patients with CML using magnetic beads with anti-CD34 antibodies (Miltenyi, no. 130-046-702) and separated on columns (Miltenyi, no. 130-042-401), following the manufacturer’s protocol. Enrichment up to ~70% of CD45^+^CD34^+^ cells was confirmed by flow cytometry analysis of the cell surface marker staining. Primary CD34^+^ cells from healthy donors were expanded in STEM SF II medium (StemSpan, no. 09655) supplemented with the essential growth factors (StemSpan, no. 02691).

### Western blotting analysis of protein level in whole-cell lysates

After treatment, the cell pellets were washed in cold PBS followed by lysis at 95°C in the SDS lysis buffer and analyzed by Western blotting, as described elsewhere (*84*). The following antibodies from Proteintech were used to detect proteins on the nitrocellulose membranes: anti-KEL (no. 67393-1-Ig), anti–c-Myc (no. 10828-1-AP), anti-NDC80 (no. 66960-1-Ig), anti-ATF4 (no. 10835-1-AP), anti-HBG1 (no. 25728-1-AP), anti-HBE1 (no. 12361-1-AP), anti-FTL (no. 10727-1-AP), anti-WDR43 (no. 30227-1-AP), anti-Gemin5 (no. 24897-1-AP), anti-RBM28 (no. 16484-1-AP), anti-RBM5 (no. 19930-1-AP), anti-SRPK1 (no. 14073-1-AP), and anti-hnRNPL (no. 18354-1-AP).

### Library preparation for long-read sequencing on the Pacific Biosciences platform

Nascent RNA was isolated from a chromatin pellet in TRIzol (Thermo Fisher Scientific, no. 15596026) after incubating at 50°C for 10 min with shaking at 1400 rpm, followed by extraction in chloroform and RNA purification using an RNeasy Mini kit (Qiagen, no. 74104). DNA was removed by on-column digestion using the RNase-Free DNase set (Qiagen, no. 79254). RNA samples were subjected to library preparation for PacBio long-read sequencing according to the procedure described before (*40*). Briefly, ribosomal RNA (rRNA) and poly(A^+^) RNA were depleted using RiboMinus Eukaryote System v2 (Thermo Fisher Scientific, no. A15026) and DynaBeads mRNA DIRECT Micropurification (Thermo Fisher Scientific, no. 61021) kits, respectively, followed by ligation of an adapter with unique molecular identifiers (UMI) to the 3′ end of RNA by T4 RNA ligase (NEB, no. M0351L), then reverse transcription by SMARTer PCR cDNA Synthesis kit (Takara/Clontech, no. 634925), and library amplification using Advantage 2 PCR kit (Takara/Clontech, no. 639137). Libraries were subjected to size selection using AMPure XP beads (Beckman Coulter, no. A63880), to select for fragments longer than 500 nt. The PacBio HiFi library was prepared using the SMRTbell Express Template Prep Kit 2.0 from PacBio following the manufacturer’s instructions. Libraries were sequenced on PacBio RS II and Revio instruments at Yale Center for Genome Discovery.

Alongside the many strengths of long-read sequencing are a few limitations, which are well known and important to acknowledge (*1*, *2*). Library preparation for long-read sequencing, including amplification by limited cycles of PCR, might bias the results toward increased splicing owing to more robust amplification of shorter reads. This is visible when comparing the fraction of spliced transcripts on the basis of reads subsampled to start at the TSS versus anywhere in the gene (e.g., Fig. 2B). To reduce this bias in the analyses focused on splicing, we included control conditions from the same library preparation as a reference. Moreover, the classification of alternative splicing events as “significant” is limited by the required level of coverage and significance. Changing cutoffs and thresholds thus modify the list of transcripts detected as “hits” in different analyses; for example, transcripts significant for a given RTI have a requirement for the indicated number of long reads (e.g., 20 or 30); this limits the number of genes that we can analyze for alternative splicing that co-occurs with readthrough.

### Nuclear and total mRNA preparation for short-read sequencing analysis

RNA from control and treated cells, whole cells [total mRNA selected for poly(A^+^) RNA during library preparation], or nuclei (nuclear RNA depleted of rRNA using RiboMinus Eukaryote System v2) obtained as an intermediate step of the cell fractionation procedure was isolated following the procedure described above. All treatments, apart from primary CML CD34^+^-enriched cells, were repeated three times as separate biological experiments. Samples were submitted for library preparation at the Yale Center for Genome Analysis core facility and sequencing analysis using Illumina NovaSeq HiSeq of paired-end 150–base pair (bp) fragments.

### Reverse transcription, real-time PCR, and Sanger sequencing

Reverse transcription of total RNA was performed using the SuperScript III Reverse Transcriptase kit (Thermo Fisher Scientific, no. 18080085), Universal Spike I RNA (TATAA Biocenter, no. RS25SI), and anchored oligo(dT_18_) (Merck, no. D2773). Reverse transcription was carried out 50°C for 60 min, followed by inactivation at 70°C for 15 min. The final cDNA solution was diluted 1:3 for chimeric junction primers and 1:10 for parental exon primers.

The junction sequence of readthrough chimeras predicted by SOAPfuse informed the design of junction-specific primers. Following reverse transcription semiquantitative polymerase chain reaction (RT-qPCR) was performed using the iTaq Universal SYBR Green Master Mix (Bio-Rad, no. 1725121) on a CFX Connect Real-Time PCR Detection System (Bio-Rad), performing 40 cycles of denaturation at 95°C for 10 s followed by annealing/extension at 60°C for 10 s. RT-qPCR amplicons were quantified using the ΔΔCt method, with universal spike-in RNA as the internal control. RT-qPCR products were electrophoresed and resolved on a 1.75% agarose gel. Bands of interest were excised and purified from the gel using the NucleoSpin Gel and PCR Clean-up kit (Takara Bio, no. 740609) according to the manufacturer’s instructions. For junction sequence validation, premixed primer and cDNA samples were sent to Quintara Bioscience for Sanger sequencing.

### Short-read sequencing analysis

#### Short-read sequencing preprocessing

The SRA Toolkit was used to download FASTQ files from the study by Bai *et al.* (*54*) (control samples: SRR29032856, SRR29032857, and SRR29032858; imatinib-resistant samples: SRR29032862, SRR29032863, and SRR29032864). For Illumina datasets generated in this study, Illumina adapters were trimmed using FASTP with the following settings: -l 15 -c --detect_adapter_for_pe --trim_poly_g --dedup --trim_front2 0 -w 15. Quality control reports for all datasets were generated using FASTQC. All Illumina datasets were mapped to the hg38 genome using the STAR (v2.7.7a) aligner with settings: --runThreadN 6 --runMode alignReads --outFilterMultimapNmax 1 --alignSJoverhangMin 8 --alignIntronMin 21 --alignIntronMax 10000 --outSAMtype BAM SortedByCoordinate. The featureCounts program (Subread/2.0.3) was used to quantify the number of reads mapping to each gene with the settings: -T 6 -p --countReadPairs -t exon -g gene_id.

#### SPI calculation

The Python tool SPLICE-q was used to quantify coverage over the 5′ and 3′ splice sites of each intron (*85*). SPI values were calculated on the basis of the SPLICE-q outputs by dividing the number of spliced counts (sum of split coverage over 5′ and 3′ splice sites) by the total number of counts (sum of unsplit and split coverage over 5′ and 3′ splice sites) mapping to that intron. To identify introns with significant changes in splicing, chi-square tests were performed by comparing the number of spliced reads and the number of unspliced reads for pairs of replicates (i.e., replicate one of untreated versus replicate one of treated, etc.). A single, adjusted *P* value for each intron was determined using the Benjamini-Hochberg, or false discovery rate (FDR), procedure. To be classified as significant, the adjusted *P* value had to be less than 0.01. To be considered for SPI analysis, an intron had to have at least 500 reads pooled across all three replicates for both conditions.

#### Alternative splicing analysis

Changes in alternative splicing events for treated samples relative to the control were detected using rMATS-turbo (*86*) in a Conda environment with the following settings: python = 3.7 rmats.py -t paired --readLength 150 --variable-read-length --novelSS --nthread 6. To be classified as significant, a change in an alternative splicing event had to have an absolute value of inclusion level difference greater than 0.1 and an adjusted *P* value less than or equal to 0.05. A read count cutoff was also implemented to keep only events with at least 20 inclusion and skipping reads across per condition, pooled across all three replicates in case of K562 cells, or across untreated and IM 1h–treated samples per patient in case of CML CD34^+^ primary cells. Only junction reads were considered in the analysis of alternative splicing events. Rmats2sashimiplot was used to quantify and visualize selected changes in splicing for particular genes.

#### Gene expression analysis with DESeq2

Differential gene expression analysis was conducted using RStudio (v4.3.1) and the R/Bioconductor package DESeq2 (v1.40.2). A variance stabilizing transformation was applied using the vst function (with the blind argument set to FALSE). Genes were classified as differentially expressed between control and treated cells if they exhibited a log_2_(fold change) greater than 0.6 (up-regulated) or lower than −0.6 (down-regulated) with an adjusted *P* value less than 0.05.

#### Gene Ontology term enrichment analysis

Functional annotation analysis of selected genes was performed using g:GOSt [g:Profiler with the Research Resource Identifier (RRID): SCR_006809, https://biit.cs.ut.ee/gprofiler/gost] with Benjamin-Hochberg FDR set to 0.05. Annotation enrichment was analyzed using Panther (www.pantherdb.org) with Fisher’s exact test requiring *P* < 0.05. Analysis of protein network enrichment was performed by searching in the ClueGO (RRID: SCR_005748) with GeneOntology (Nov2024) at CytoScape (v3.10.3) (RRID: SCR_003032) with Benjamini-Hochberg FDR set to 0.05.

#### Identification of readthrough chimera transcripts with SOAPfuse

RNA-seq data were preprocessed using FASTP and mapped to the hg38 genome using the STAR aligner with settings: --runThreadN 16 --runMode alignReads --outFilterMultimapNmax 1 --alignSJoverhangMin 1 --alignIntronMin 21 --alignIntronMax 200000 --readFilesCommand zcat --outSAMtype BAM SortedByCoordinate --outFileNamePrefix ../results/bam/ --sjdbGTFfile {input.gtf}--genomeDir {input.genome_dir} --readFilesIn {input.R1_P} {input.R2_P} --chimOutType WithinBAM Junctions --chimMultimapNmax 2 --chimSegmentMin 15 --chimOutJunctionFormat 1 --chimMultimapScoreRange 3 --chimJunctionOverhangMin 15 --chimScoreJunctionNonGTAG -4 --chimNonchimScoreDropMin 10 --alignMatesGapMax 200000 --chimScoreSeparation 5 --chimScoreMin 0. Resulting bam files were used for alternative splicing analysis by rmats2Sashimiplot.

SOAPfuse (v1.26) was used to detect fusion transcripts from preprocessed paired-end RNA-seq data (*87*). Alignment database was built using the hg38 human genome and gene annotation files from ENCODE. Reads pooled from biological replicates of the same condition were aligned to the database using the the Burrows-Wheeler aligner. SOAPfuse analysis settings required minimum three span reads and three junction covering reads, and a minimal intrachromosome distance between genes of 500 nt. For comparative analysis, the output list was filtered to include only fusions with at least a total of six supporting reads and removed any fusions with ribosomal, small nucleolar, and small nuclear RNAs. To specifically focus on potential readthrough chimeras in each RNA-seq dataset, only chimeras between adjacent genes sharing the same strand and orientation were considered.

### Long-read sequencing analysis

#### Long-read sequencing preprocessing

Adapter sequences were removed using cutadapt (v3.4) with the following settings: cutadapt --cores = 8 --discard-untrimmed --no-indels --revcomp -e 0.15. Long reads were mapped to the GRCh38.p14/hg38 genome (Ensembl) using minimap2 (v2.22) with settings -ax splice:hq –secondary = no -t 12 -a -u f –seed 14. SAM outputs from minimap2 were converted to BAM and BED files using SAMtools (v1.21) and BEDTools (v2.30.0), respectively. UMICollapse (v1.0.0) was used for deduplication with the following settings: umicollapse -Xmx60g bam --umi-sep. Polyadenylated reads and nonunique reads (i.e., entries with duplicate read names) were removed using a custom script. Preprocessing steps and custom scripts to filter poly(A)^+^ reads were consolidated in Snakemake.

#### Genome annotation filtering

The “Convert GTF to BED12” feature of the Galaxy web server (https://usegalaxy.org/) was used to generate a BED12 version of the hg38 genome with entries for all transcripts. For splicing analysis, transcript entries were filtered to keep only the canonical transcript as defined by Ensembl for each gene. For readthrough analysis, the transcript with the distal-most 3′ end was used to prevent false-positive readthrough identification.

#### Comparison of gene coverage

Analysis of gene region coverage was performed using deepTools2 (*88*) in a Conda environment. First, bamCoverage tool was used with the following options: --binSize 40 --normalizeUsing RPKM --effectiveGenomeSize 2913022398. Coverage from three replicates was averaged using bigwigAverage. The resulting bigWig files were used to compare coverage of reads within the gene body and 4 kb before the annotated TSS or after the annotated transcription end site. A coverage matrix was prepared using computeMatrix scale regions with the following settings: -R hg38_genes.bed (a reference containing only protein-coding genes was used) -bs 40 -b 4000 -a 4000 --regionBodyLength 7000 --skipZeros --missingDataAsZero. For plotting, the coverage traces were aligned within the gene body region.

#### Splicing status classification and CoSE calculation

Splicing status classification and CoSE calculation were performed as described (*6*). The splicing status of each long read was assigned on the basis of the number of spliced introns it contained relative to the total number of introns it overlapped. Reads for which all possible introns were removed were classified as “all spliced,” reads for which all possible introns were not yet removed were classified as “all unspliced,” and reads with a combination of spliced and unspliced introns were classified as “partially spliced.” CoSE values were calculated for all introns from canonical transcripts. CoSE represents the number of spliced reads spanning an intron divided by the total number of reads spanning an intron.

#### Readthrough status classification and RTI calculation

Long reads were intersected with the last exon of the representative transcript for each gene using bedtools. For reads overlapping multiple genes, only the overlap with the greatest coverage was used for analysis. For each gene, the readthrough boundary was defined as 100 nt downstream of the PAS. A read was classified as “readthrough” if its 3′ end extended beyond the readthrough boundary or classified as gene body if its 3′ end did not extend beyond the readthrough boundary. The RTI was calculated for each gene by dividing the number of readthrough reads by the total number of reads mapping to the last exon of the gene. To be classified as a readthrough gene, the RTI had to be greater than or equal to 0.2.

The controls used for splicing analysis (CoSE, percentage of spliced reads) represent the control samples prepared concurrently with the imatinib-treated samples (GSM8673083, GSM8673084, and GSM8673085); selecting this subset of controls corrects for potential differences in library preparation. The controls used for comparison of CoSE versus RTI values represent all control samples (GSM8673080, GSM8673081, GSM8673082, GSM8673083, GSM8673084, and GSM8673085).

#### PAS coverage calculation

Long-read coverage in the region downstream of PASs was quantified using a custom Python script. Coverage was normalized to the position 200 nt upstream of each PAS.
