# Supplementary material for: Transcriptional readthrough precedes alternative splicing programs triggered in CML cells by imatinib
Source: Sci Adv. 2026 Mar 20;12(12):eaea2475. doi: 10.1126/sciadv.aea2475 (PMC13004010; doi:10.1126/sciadv.aea2475)
Supplement: Supplementary file 1 — Figs. S1 to S10 Tables S1 to S4 [file sciadv.aea2475_sm.pdf]

Science Advances
AAAS

Supplementary Materials for

Transcriptional readthrough precedes alternative splicing programs triggered in CML cells by imatinib

Paulina Podszywałow-Bartnicka *et al.*

Corresponding author: Paulina Podszywałow-Bartnicka, paulina.podszywalow-bartnicka.@yale.edu;
Karla M. Neugebauer, karla.neugebauer@yale.edu

Sci. Adv. **12**, eaea2475 (2026)
DOI: 10.1126/sciadv.aea2475

This PDF file includes:

Figs. S1 to S10
Tables S1 to S4

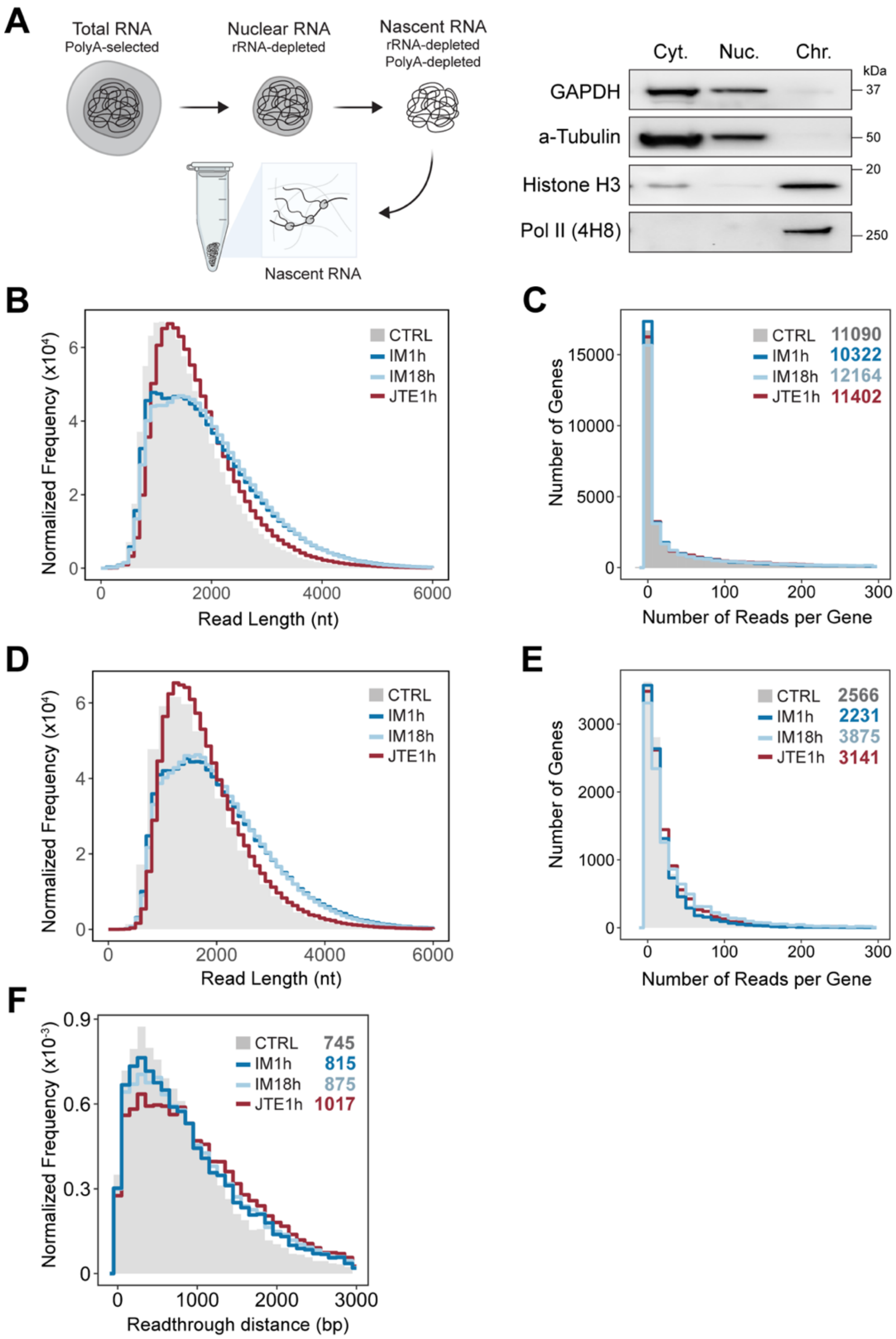

Figure S1. Preparation and properties of long-read sequencing datasets.

(A) Schematic showing the steps involved in purifying nascent RNA from the chromatin fraction (left). Representative image of western blotting analysis of protein level in cell fractions (Cyt. – cytoplasm; Nuc. – nucleus; Chr. – chromatin) obtained during isolation of nascent RNA from the chromatin fraction (right). Analyses in **(B, C)** include all long reads after poly(A) filtering and splicing intermediate removal, while analyses in **(D-F)** include only filtered long reads that overlap with the last exon of their gene of origin. **(B, D)** Read length distributions normalized to the number of reads in each condition. **(C, E)** Read depth distributions. The number of genes with at least 30 reads **(C)** mapping anywhere along the gene or **(E)** mapping to at least the last exon are provided at the top right. **(F)** Distribution of readthrough distances for expressed genes measured beginning 100 nt after the poly(A) signal of the longest annotated isoform. The median readthrough distance for each condition is provided at the top right.
See Table S1 for numerical data.

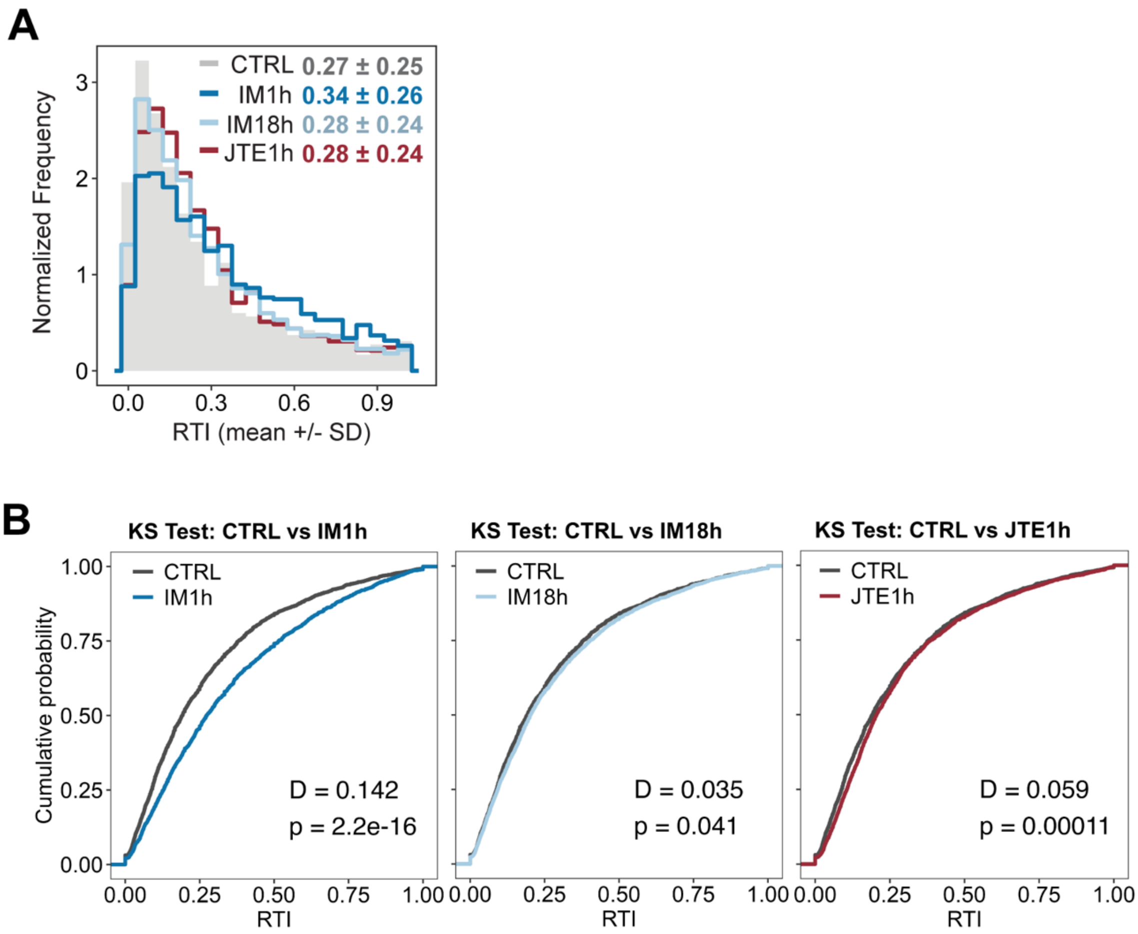

Figure S2. Readthrough transcription index (RTI) score analysis for long-read sequencing data.

(A) Distribution of RTI values per gene for each condition. RTI values (mean ± s.d.) were calculated based on 3 biological replicates per condition and are provided in the top right corner of the plot. **(B)** Comparison of the cumulative probability distribution of RTI scores in CTRL versus IM1h, IM18h or JTE1h treated samples by Kolmogorov-Smirnov test. Values of maximum absolute difference (D) and its significance (p) are provided under the curve. Number of genes included in the analysis: 2566 in CTRL; 2231 in IM1h; 3875 in IM18h; 3141 in JTE1h.

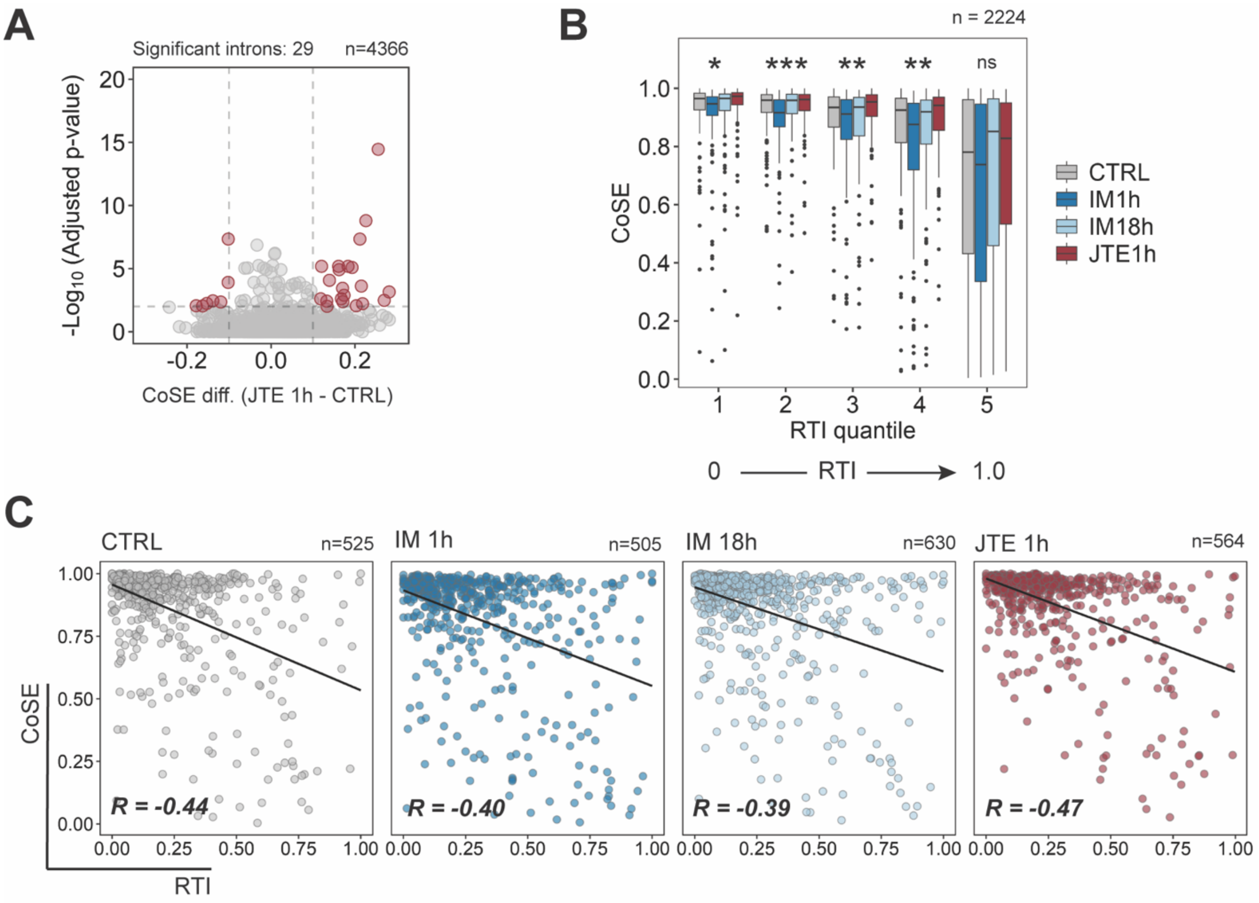

Figure S3. Analysis of co-transcriptional splicing efficiency (CoSE) in K562 cells before and after treatments.

(A) Change in CoSE value for 1h JTE-treated cells relative to CTRL cells plotted for individual introns. Introns that undergo significant changes in splicing efficiency (|Change in CoSE| ≥ 0.1 and adjusted p-value < 0.05) are marked in red. Chi-square tests were performed by comparing the number of spliced reads and the number of unspliced reads for pairs of replicates (i.e., replicate one of untreated versus replicate one of treated, etc.). A single, adjusted p-value for each intron was determined using the Benjamini-Hochberg, or False-Discovery Rate, procedure. Number of introns analyzed in the top right corner (n). **(B-C)** Comparison of CoSE versus RTI. Only genes meeting threshold coverage for RTI and CoSE analysis are included in the comparison (n - number of genes). **(B)** Analysis of CoSE value within each RTI quantile that corresponds to Fig.1D. One-way ANOVA with Dunnett's test used to compare IM1h to other conditions within the quantile; ***p<0.0002, **p<0.005, *p<0.03, ns p>0.05. **(C)** Scatter plots depicting the relationship between RTI and the average CoSE value for each gene analyzed; R – correlation coefficient value.

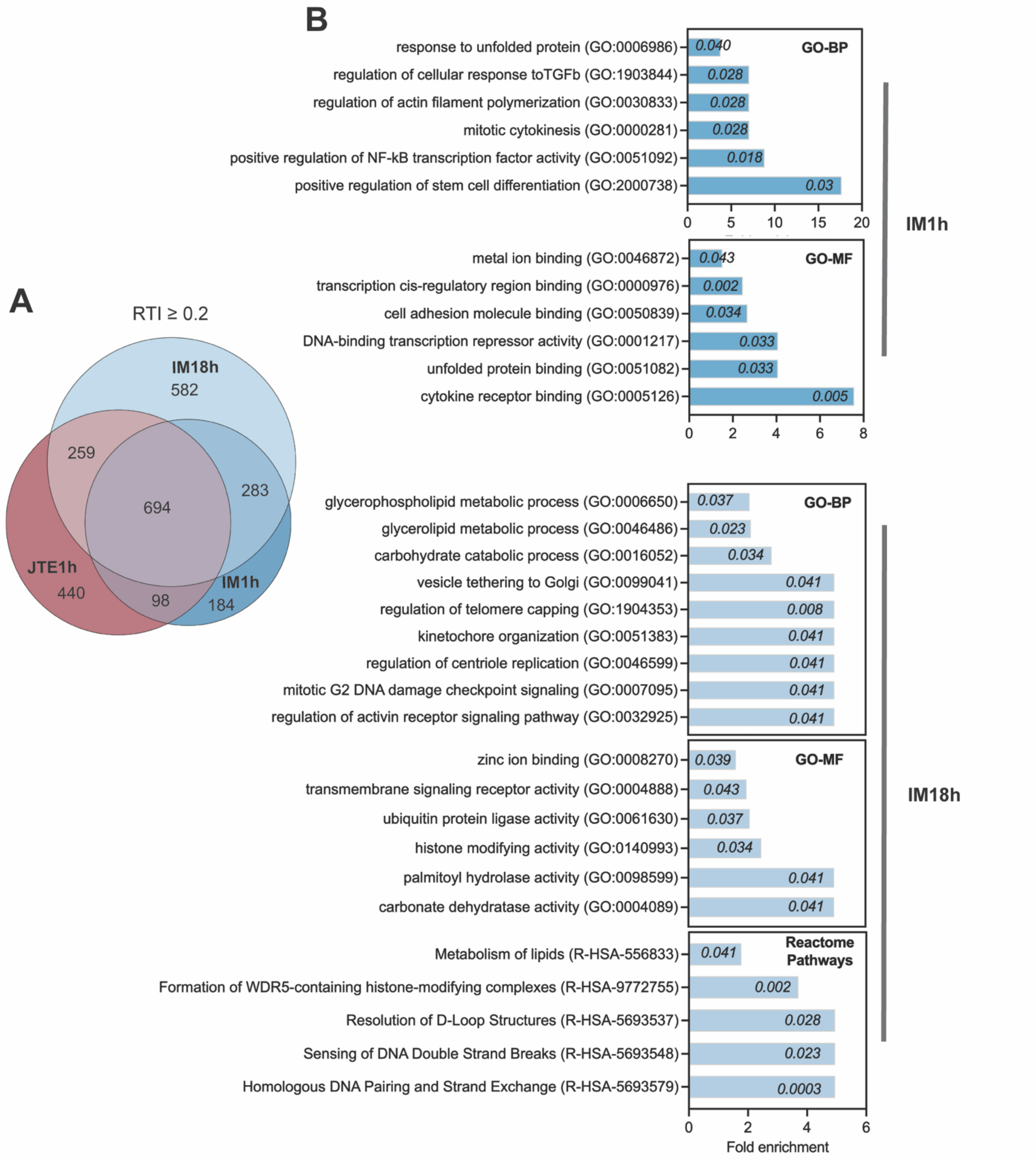

Figure S4. Gene Ontology terms enrichment analysis of genes with high transcriptional readthrough after IM 1h and 18h treatments

(A) Comparison of genes with RTI ≥ 0.2 that have at least 30 reads across 3 replicates in each condition. IM – imatinib (1818 of genes in IM18h and 1259 of genes in IM1h), JTE – JTE-607 (1491 of genes). **(B)** Gene Ontology Biological Processes (GO-BP), Molecular Function (MF), and Reactome Pathways terms enrichment analysis of genes from the intersection of RTI ≥ 0.2 (in A) that, excluding the ones that have RTI ≥ 0.2 in CTRL and JTE, are unique to IM 1h (132 genes) or IM 18h (616 genes); Fisher's exact test fold enrichment to all RTI ≥ 0.2 genes identified displayed and FDR value provided in the bars.

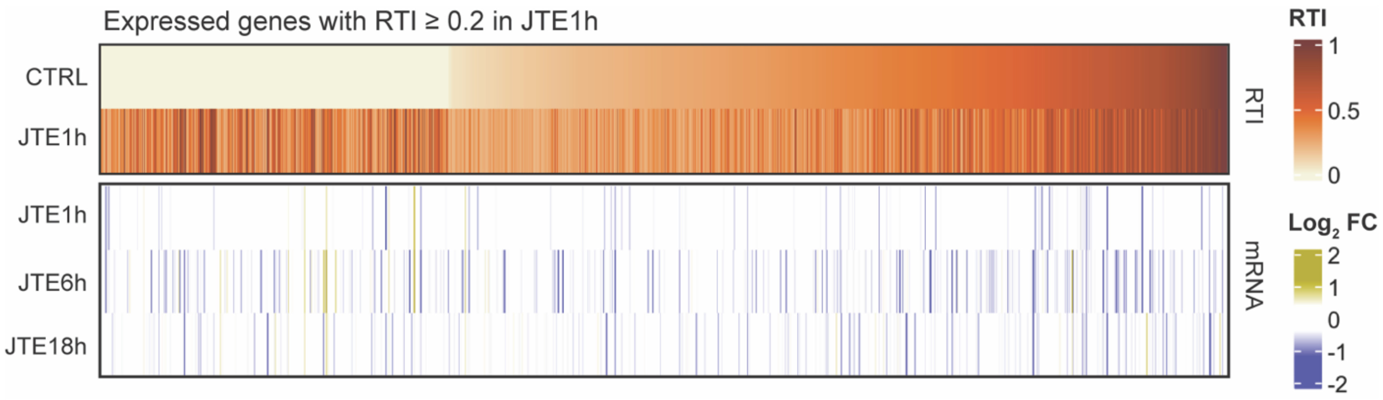

Figure S5. Changes in gene transcriptional readthrough and expression upon JTE treatment.

Changes in RTI and mRNA levels upon JTE 1h treatment for expressed genes with RTI ≥ 0.2 (n=1491), ordered by RTI in CTRL. Log2FC - log2 of fold change relative to CTRL.

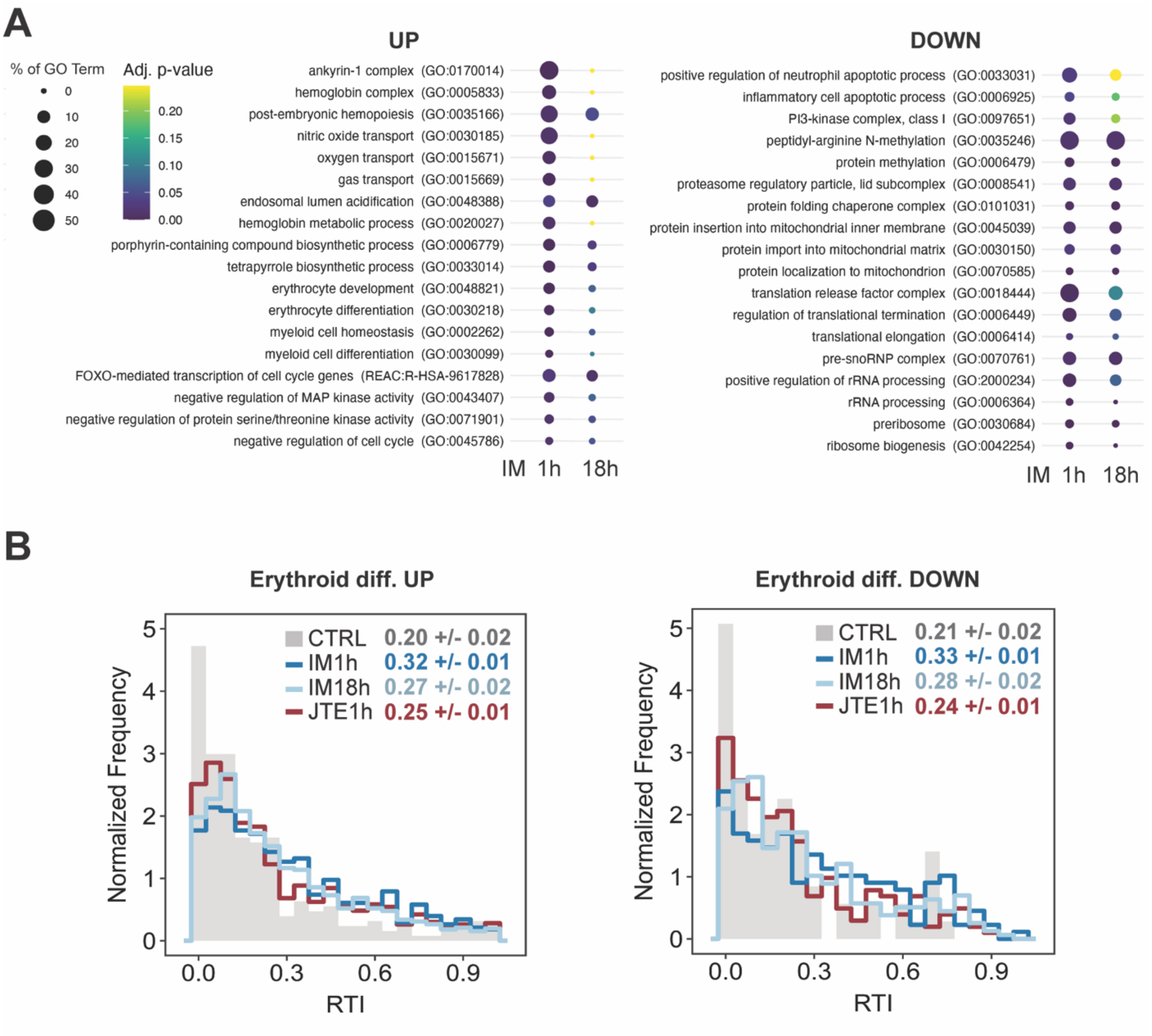

Figure S6. Transcriptional readthrough in the context of cell response to treatment.

(A) Gene Ontology Biological Processes terms enrichment analysis of genes with RTI ≥ 0.2 in IM18h or IM1h and RTI difference to CTRL ≥ 0.2 that at the mRNA level in IM18h are upregulated (UP, Log2FC ≥ 0.6) or downregulated (DOWN, Log2FC ≤ -0.6) relative to CTRL. Circle size - percentage of genes representing given term (% of GO Term); Fisher exact test with Bonferroni-Hochberg correction used to estimate significance (Adj. p-value). **(B)** Frequency histograms showing transcriptional readthrough index (RTI) values in genes that are differentially expressed upon induction of erythroid differentiation in (An et al. 2014).

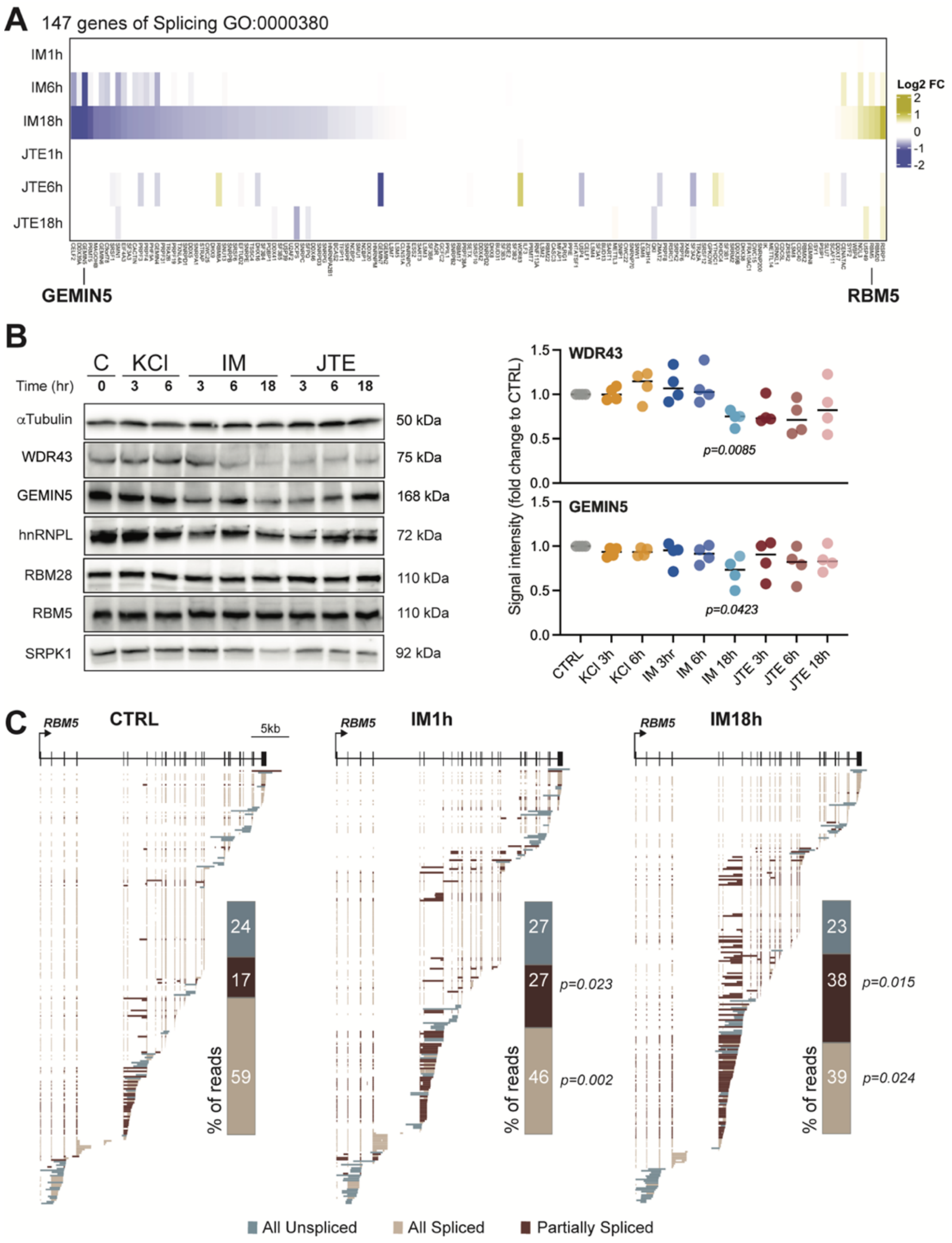

Figure S7. Imatinib affects the expression of proteins involved in the regulation of splicing.

(A) Differential expression of human genes (n=147) that are annotated in QuickGO to the term 'Splicing' (GO:0000380, including dependent terms, relationship set to 'is_a', 'part_of', 'occurs_in'; and with experimental evidence ECO:0000269), ordered by value in IM18h of log2 of fold change relative to CTRL (Log2FC). **(B)** Western blot detection of proteins encoded by selected up- and down-regulated genes important in RNA processing and splicing. Left panel - representative images; equal amounts of protein from whole cell lysates were loaded in each lane; αTubulin for reference. Right panel – bands signal intensity in immunoblot detection of proteins WD Repeat Domain 43 (WDR4) and Gem-associated protein 5 (Gemin5), measured by densitometry and presented as fold change to CTRL; results from experiment replicates represented by a dot (n=4). **(C)** Increased intron retention in nascent RNAs mapping to RNA Binding Motif Protein 5 gene (*RBM5*). Nascent RNA long reads downsampled to 250, colored according to splicing status; mean number of reads in each category presented in the bars (n=3). **(B-C)** Student's two-way t-test was used to estimate the significance of change (p) upon IM 18h or IM 1h to CTRL.

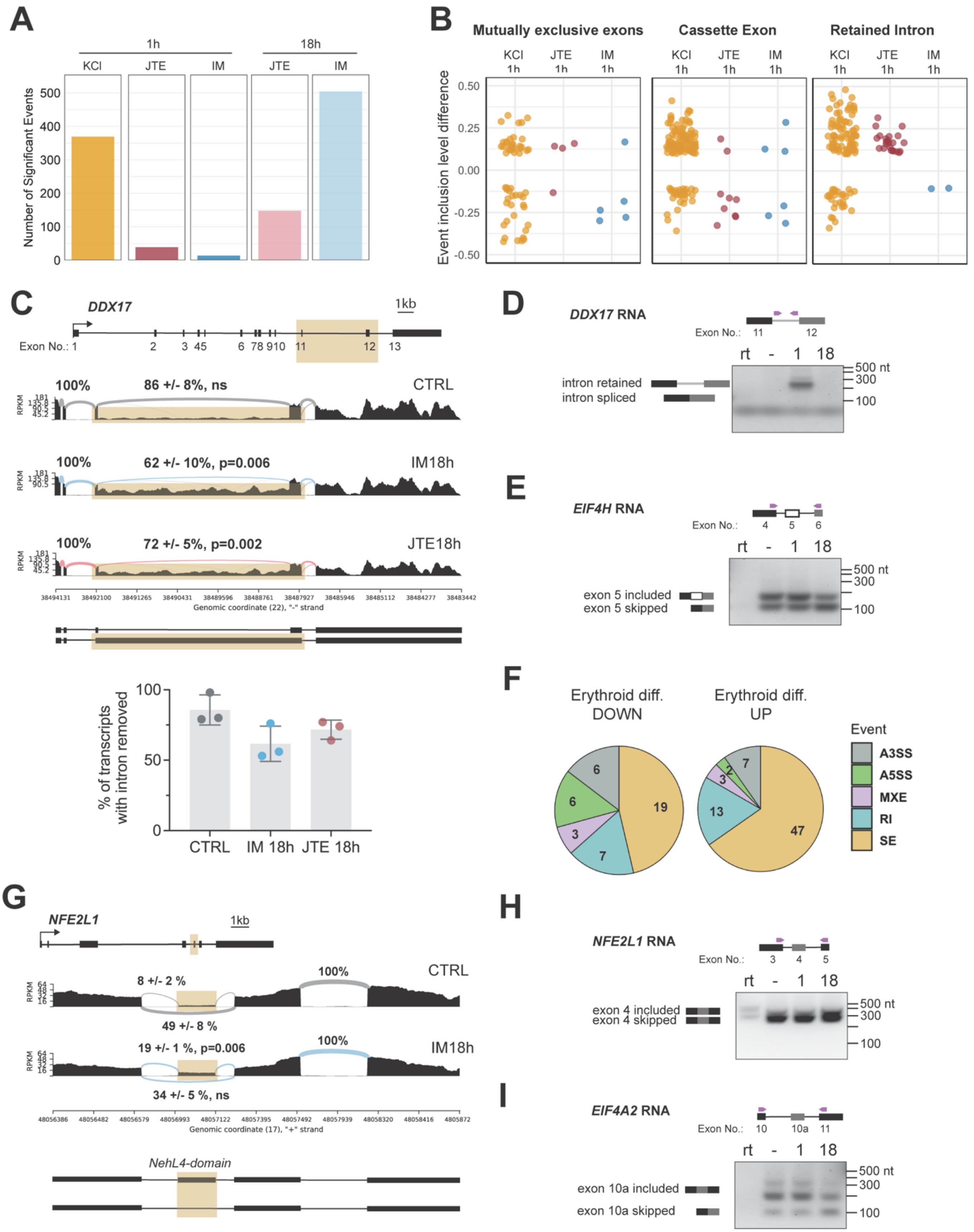

Figure S8. Impact of treatment on alternative splicing (legend on the next page).

Figure S8. Impact of treatment on alternative splicing (legend on the next page).

(A) Number of significant (FDR<0.05, 20 reads per event) alternative splicing events in each treatment through analysis of mRNAseq. **(B)** Level difference of exon/intron inclusion (> 0.1) or exclusion (< -0.1); only significant events detected by mRNAseq. **(C)** Sashimi plot showing splicing of DEAD-box RNA helicase 17 (*DDX17*) that is characterized by RTI≥0.2 and intron retention upon IM18h relative to CTRL. Upper panel - coverage and splicing analysis as % of upstream intron on the right; Lower panel – mean value of percent isoforms with intron removed (mRNAseq, n=3). (**D-E**) Images of RT-PCR products from whole cells untreated (-), imatinib-treated cells for 1h or 18h, and no reverse transcriptase control (rt), resolved in an agarose gel for the detection of alternative splicing events by fluorescent staining. Primers (purple) binding indicated above scheme presenting a fragment of DDX17 **(D)**, EIF4H **(E)**. **(F)** Alternative splicing events detected in nuclear RNA with significant differences in IM18h relative to CTRL, considering genes identified as UP/DOWN regulated upon CD34+ erythroid differentiation; number of events provided in the piechart. **(G)** Alternative splicing analysis of mRNAseq reads mapping to Endoplasmic reticulum membrane sensor NFE2L1, precursor of transcription factor NRF1 (*NFE2L1*). (**H-I**) Detection of alternative splicing of exon encoding for Nhe4l domain in NFE2L1 (**H**) and poison cassette exon in EIF4A2 **(I)** in by RT-PCR (see above).

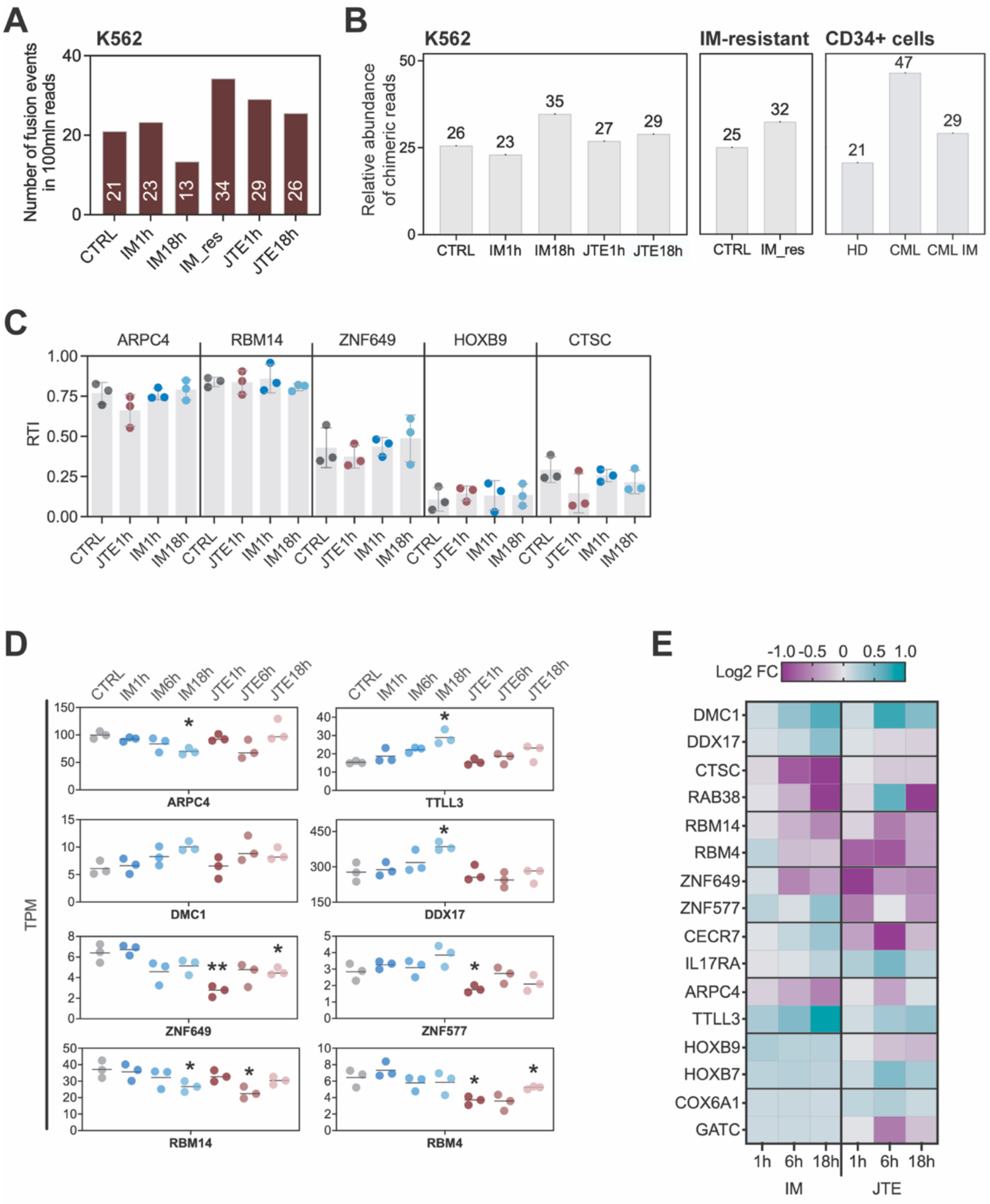

Figure S9. Detection of chimeric transcripts by analysis of short-read mRNA -seq data

(A) Number of readthrough fusion-supporting reads per readthrough chimera in each RNA-seq dataset identified by SoapFuse analysis of short-read sequencing of mRNA from K562 and CD34+ enriched CML cells from patients, untreated (CTRL) or treated with imatinib (IM) or JTE-607 (JTE) for 1h or 18h. Included are also results from analysis of publicly available data from K562 cells imatinib-resistant (Bai J et al., 2024, GSE267522). **(B)** Average number of reads covering fusion per readthrough chimera. HD – data from CD34+ enriched cells from blood samples of 6 healthy donors (Pellagatti A et al., 2018, GSE114922). CML – our data from sequencing of mRNA from CD34+ enriched cells; CML_IM – 1h imatinib treated. **(C)** RTI value for selected genes that are upstream in chimera transcripts. **(D)** Normalized number of reads to the density of sequencing (TPM - transcripts per million reads) for upstream (left) and downstream (right) genes included in the selected chimeric transcripts. Circle - biological replicate; black line – mean value of 3 biological experiments. Student's t-test was used to estimate significance of change upon IM18h, JTE1h or JTE18h to CTRL; * p<0.05, ** p<0.005. **(E)** Fold change of mRNA level (FC) for chimera forming up- and downstream genes in IM and JTE-treated cells for 1, 6 and 18h relative to CTRL.

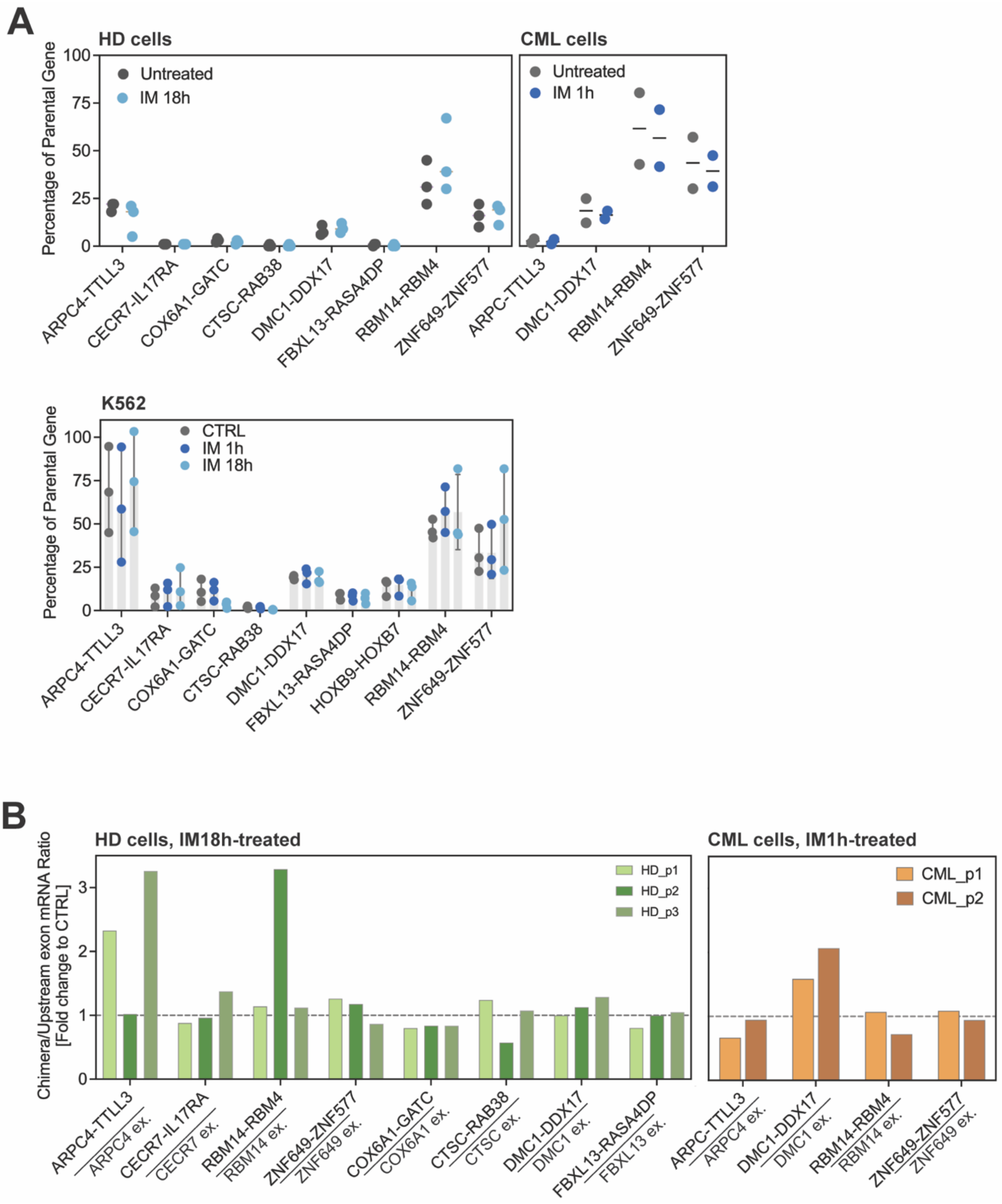

Figure S10. Analysis of chimera transcripts level by real time PCR.

(A) Upper panel – primary CD34+ enriched cells from 3 healthy donors (HD) or 2 CML patients, represented by circle, untreated or IM-treated for 18h (in HD) or 1h (in CML). Data normalized to spike-in expressed as percentage of chimera over upstream gene. **(B)** Comparative analysis of the relative change in chimera abundance upon IM treatment in mRNA from primary cells. Data normalized to spike-in and last exon in the upstream gene presented as fold change to CTRL (set as 1.0 and represented by the dashed line).

Table S1

Sample	raw reads	mapped	w/o poly(A) reads	w/o splicing intermediates
CTRL_R1	967174	416299	415587	360719
CTRL_R2	1109544	489147	488570	410442
CTRL_R3	676058	284141	283657	258592
**CTRL_R1-3 sum**	**2752776**	**1189587**	**1187814**	**1029753**
KCl 45min_R1	1084120	469860	468992	410655
KCl 45min_R2	807082	343397	342671	313838
KCl 45min_R3	2724768	1119386	1117365	1055596
**KCl_R1-3 sum**	**4615970**	**1932643**	**1929028**	**1780089**
JTE 1h_R1	1192970	519597	518931	459508
JTE 1h_R2	1391536	588866	587802	514227
JTE 1h_R3	3202118	1298220	1295465	1147511
**JTE 1h_R1-3 sum**	**5786624**	**2406683**	**2402198**	**2121246**
CTRLim_R1	296319	271399	271078	229855
CTRLim_R2	759545	658228	657402	540074
CTRLim_R3	680546	599416	598488	492306
**CTRLim_R1-3 sum**	**1736410**	**1529043**	**1526968**	**1262235**
IM 1h_R1	907968	792025	790770	673042
IM 1h_R2	785490	678843	677829	573238
IM 1h_R3	1112676	949138	947854	802732
**IM 1h_R1-3 sum**	**2806134**	**2420006**	**2416453**	**2049012**
IM 18h_R1	1185161	1036352	1034219	939959
IM 18h_R2	1709691	1441933	1439745	1277422
IM 18h_R3	1755428	1510965	1508618	1349556
**IM 18h_R1-3 sum**	**4650280**	**3989250**	**3982582**	**3566937**
**TOTAL**	**22348194**	**13467212**	**13445043**	**11809272**

Table S1. Long-read RNA sequencing datasets and their mapping statistics. Number of raw reads, mapped reads, poly(A) and splicing intermediates filtered reads from long-read sequencing of nascent RNA (Pacific Biosciences platform) for each described sample. R, biological replicate.

Table S2

	All reads mapped to protein-coding genes								Gene body reads		Readthrough reads	
	All		Spliced		Unspliced		Partially spliced		All		All	
Sample	Median	SD	Median	SD	Median	SD	Median	SD	Median	SD	Median	SD
CTRL1	1409	565	1357	536	1459	587	1551	607	1400	558	1685	672
CTRL2	1427	575	1362	540	1501	602	1581	609	1419	569	1743	661
CTRL3	1711	805	1530	683	1888	850	1967	895	1693	792	2187	948
KCL1	1390	549	1352	526	1427	571	1551	593	1383	544	1678	654
KCL2	1711	840	1586	758	1833	892	1965	915	1699	831	2144	1000
KCL3	1552	726	1461	657	1672	782	1790	807	1543	718	1952	893
JTE1	1313	506	1281	486	1379	541	1496	555	1307	501	1588	618
JTE2	1813	870	1650	781	1939	905	2034	926	1784	847	2320	1032
JTE3	1694	801	1573	720	1837	856	1939	879	1669	777	2243	991
IM1h1	1871	977	1613	874	2068	1004	2143	1024	1838	961	2533	1066
IM1h2	1798	969	1542	855	2009	1003	2087	1014	1766	953	2464	1068
IM1h3	1836	981	1574	868	2044	1015	2116	1023	1804	965	2519	1071
IM18h1	1915	949	1703	864	2131	983	2211	990	1891	937	2512	1038
IM18h2	1782	943	1592	852	1998	988	2083	994	1756	929	2434	1041
IM18h3	1862	946	1636	851	2067	981	2156	979	1837	935	2438	1028

Table S2. Read length characteristics of each long-read dataset. Median with standard deviation (SD) values are shown in nucleotides. The reads described were filtered according to the splicing status or alignment region: Spliced - all introns spliced out, Unspliced - all introns present, Partially spliced - at least one intron spliced out and one intron present, Gene body reads - align within the region of the gene, Readthrough reads - align within the region of the gene covering at least the last exon and extend to the downstream of gene region.

Table S3

Sample	Read status			
	Sequenced	Mapped	Unassigned_ NoFeatures	Unassigned_ Ambiguity
Sequencing of mRNA				
CTRL_R1	22249243	19031221	1719706	1498316
CTRL_R2	21683902	18333127	1875962	1474813
CTRL_R3	20876632	17855236	1610102	1411294
**CTRL_R1-3 sum**	**64809777**	**55219584**	**5205770**	**4384423**
IM 1h_R1	22524078	18816370	2214106	1493602
IM 1h_R2	25663644	21756583	2153436	1753625
IM 1h_R3	25359082	21655845	2011048	1692189
**IM 1h_R1-3 sum**	**73546804**	**62228798**	**6378590**	**4939416**
IM 6h_R1	26511693	22054838	2715165	1741690
IM 6h_R2	22637221	19068166	2009553	1559502
IM 6h_R3	20873266	17578358	1908664	1386244
**IM 6h_R1-3 sum**	**70022180**	**58701362**	**6633382**	**4687436**
IM 18h_R1	21832167	18232489	2103651	1496027
IM 18h_R2	22563898	18420740	2629794	1513364
IM 18h_R3	21269043	17661189	2168839	1439015
**IM 18h_R1-3 sum**	**65665108**	**54314418**	**6902284**	**4448406**
JTE 1h_R1	22303801	19219839	1553035	1530927
JTE 1h_R2	23519895	20323444	1543082	1653369
JTE 1h_R3	22164714	19222008	1421033	1521673
**JTE 1h_R1-3 sum**	**67988410**	**58765291**	**4517150**	**4705969**
JTE 6h_R1	20133094	16971725	1846229	1315140
JTE 6h_R2	22011839	18666920	1853861	1491058
JTE 6h_R3	25567472	21826935	2027655	1712882
**JTE 6h_R1-3 sum**	**67712405**	**57465580**	**5727745**	**4519080**
JTE 18h_R1	21920550	18460064	2036704	1423782
JTE 18h_R2	19262757	16439848	1502147	1320762
JTE 18h_R3	21378678	18095868	1884358	1398452
**JTE 18h_R1-3 sum**	**62561985**	**52995780**	**5423209**	**4142996**
KCl 1h_R1	22217976	19270518	1416170	1531288
KCl 1h_R2	72943502	62953314	5081974	4908214
KCl 1h_R3	49312141	42474454	3514237	3323450
**KCl 1h_R1-3 sum**	**144473619**	**124698286**	**10012381**	**9762952**
KCl 3h_R1	24077102	20822856	1573823	1680423
KCl 3h_R2	26004252	22505595	1662814	1835843
KCl 3h_R3	23313941	20242385	1464866	1606690
**KCl 3h_R1-3 sum**	**73395295**	**63570836**	**4701503**	**5122956**
**TOTAL**	**1380351166**	**1175919870**	**111004028**	**93427268**

Sample	Read status			
	Sequenced	Mapped	Unassigned_ NoFeatures	Unassigned_ Ambiguity
Sequencing of nuclear RNA				
CTRL_R1	33461963	22588484	10876630	2061588
CTRL_R2	32749339	22272506	10368629	2213696
CTRL_R3	30988231	19803825	11211153	1967445
**CTRL_R1-3 sum**	**97199533**	**64664815**	**32456412**	**6242729**
IM 1h_R1	28385612	20421694	7869067	1858137
IM 1h_R2	34601349	22828792	11745409	2162499
IM 1h_R3	30044816	20924712	8990724	2052763
**IM 1h_R1-3 sum**	**93031777**	**64175198**	**28605200**	**6073399**
IM 18h_R1	36035776	23580149	12268138	2300791
IM 18h_R2	30929803	20075848	10693243	2036782
IM 18h_R3	25304023	16175394	9014972	1682168
**IM 18h_R1-3 sum**	**92269602**	**59831391**	**31976353**	**6019741**
JTE 1h_R1	35284411	23709633	11556796	2157487
JTE 1h_R2	30225874	20855304	9261584	1981171
JTE 1h_R3	36189754	25103205	10927819	2480259
**JTE 1h_R1-3 sum**	**101700039**	**69668142**	**31746199**	**6618917**
KCl 1h_R1	28121738	19251170	8806106	1758029
KCl 1h_R2	31773769	21728391	9939890	2129214
KCl 1h_R3	32496131	20518683	11971735	2040885
**KCl 1h_R1-3 sum**	**92391638**	**61498244**	**30717731**	**5928128**
**TOTAL**	**953185178**	**639675580**	**311003790**	**61765828**

Table S3. Short-read RNA sequencing and mapping statistics. Number of raw reads sequenced on the Illumina platform, counts of mapped reads, number after filtering out the reads mapped to a region that is not annotated in hg38 annotation (Unassigned_NoFeatures), Number after filtering for reads mapped to multiple locations in hg38 reference genome (Unassigned_ambiguity). R, biological replicate.

Table S4

Chimeric Junction	Sequence	Amplicon Size
ARPC4-TTLL3_F	GAAGCTGTCAGTCAATGCCC	
ARPC4-TTLL3_R	TCTGAGCCGGTTCATGTGAG	**111**
CECR7-IL17RA_F	GGACCAGCTGACTAAGGAGC	
CECR7-IL17RA_R	CTTGTTGGGTGTGGGCAAAG	**177**
COX6A1-GATC_F	TGTGTACCTGAAGTCGCACC	
COX6A1-GATC_R	CGATAGCTTTCTCCAGTCGC	**184**
CTSC-RAB38_F	CAGACCCCAATCCTAAGCCC	
CTSC-RAB38_R	ATGCACCCATAGCTTCTCGG	**112**
DMC1-DDX17_F	AGCTTGCGAAAGGGAAGAGG	
DMC1-DDX17_R	TGTCAGCCTTGCTACTTCCG	**198**
FBXL13-RASA4DP_F	GCTGCAAACAACTCCGGATC	
FBXL13-RASA4DP_R	TGTGTCTGTGCTACTGTCTTCC	**133**
HOXB9-HOXB7_F	CAGTTTGGAAACTTCGGCGG	
HOXB9-HOXB7_R	TCTGGTAGCGGGTGTAGGTC	**155**
RBM14-RBM4_F	TTTTCGTGGGCAATGTGTCG	
RBM14-RBM4_R	TCACATTCCAGCACCTTCCC	**203**
ZNF649-ZNF577_F	ACATCACAGAACTCACACGGG	
ZNF649-ZNF577_R	AGCCTGGAAGCTCCTTCTTC	**103**

Upstream Parental Exon	Sequence	Amplicon Size
ARPC4_Ex5_F	ACTTCCACACAGAGCAGATGT	
ARPC4_Ex5_R	TCTTCAGCCACAATGCGGG	**130**
CECR7_Ex1_F	AGAGAACACAGCCGAAGTGG	
CECR7_Ex1_R	CCAGGAACCAGATGTAGCCC	**89**
COX6A1_Ex2_F	AGCTCGCATGTGGAAGACTC	
COX6A1_Ex2_R	TGGTGCGACTTCAGGTACAC	**87**
CTSC_Ex5_F	CAAACCTGCACCACTGACTG	
CTSC_Ex5_R	CTTGGTTTCGAACAGGACTGAC	**113**
DMC1_Ex_F	ACCCATTGGGGGACACATTC	
DMC1_Ex_R	GCTCTCCTCTTCCCTTTCGC	**71**
FBXL13_Ex_F	CAGCAATGGAGATGTTATCGGC	
FBXL13_Ex_R	GATCCGGAGTTGTTTGCAGC	**122**
HOXB9_Ex1_F	CAGTTTGGAAACTTCGGCGG	
HOXB9_Ex2_R	GATCCGGCCTCTCTTTGTCC	**107**
RBM14_Ex1_F	GAAACAGTTCGCCTTCGTGC	
RBM14_Ex1_R	CGACACATTGCCCACGAAAA	**160**
ZNF649_Ex1_F	CCAGTGCCGGGGTTTAAGG	
ZNF649_Ex1_R	CTGATTTTCTTCTGGCTACACCT	**70**

Alternative splicing validation	Sequence	Amplicon size
EIF4H_Ex4_F	GTGTGGACATTGCAGAAGGC	
EIF4H_Ex6_R	GCCCCCTAAGAAGTCATCCC	**101**
DDX17_Ex11_F	CTGGAAAGGCACCCATCCTT	
DDX17_Ex12_R	CCTCTGAGCTGTTTGGATAGTCA	**105**
NFE2L1_Ex3_F	CTGATTGACATCCTTTGGCGAC	
NFE2L1_Ex5_R	GGCTCACTCTCACTAGGCAC	**257**
EIF4A2_Ex10_F	ATGTGCAACAAGTGTCTTTGGT	
EIF4A2_Ex11_R	CTCCCAAATCGACCCCCTCT	**94**

Downstream Parental Exon	Sequence	Amplicon Size
TTLL3_Ex_F	ACAATCCAAGGCTGCTACCC	
TTLL3_Ex_R	CCCATCGCTGAGCTATCCAG	**131**
DDX17_Ex2_F	TTTGGTAATCCTGGGGAGCG	
DDX17_Ex2_R	TGTCAGCCTTGCTACTTCCG	**108**
RBM4_Ex2_F	TGGGAAGGTGCTGGAATGTG	
RBM4_Ex2_R	GACTGATGTTGCCCACATGC	**185**
ZNF577_Ex_F	CCAGTGGTAGCTTGACCTGA	
ZNF577_Ex_R	GGCCTTGCCCATTTCGTTCA	**123**

Readthrough validation	Sequence	Amplicon size
HBE1_Ultimate exon_F	CTGGGTAACGTGATGGTGATTA	
HBE1_Readthrough_R	CCTAGGAGTCTCTGTCCACTTA	**488**
FTH1_Ultimate exon_F	GAATCTGGCTTGGCGGAATA	
FTH1_Readthrough_R	GGTGGTTGCAGTGAGATTGA	**847**
HBE1_Ex1_F	ATGGTGCATTTTACTGCTGAGG	
HBE1_Ex2_R	GGGAGACGACAGGTTTCCAAA	**156**
FTH1_Ex1_F	TCCTACGTTTACCTGTCCATGT	
FTH1_Ex2_R	GTTTGTGCAGTTCCAGTAGTGA	**268**

Table S4. Primers designed for real-time PCR analysis of transcript level and alternative splicing.
F - forward, R - reverse, Ex – exon.
